# Effects of drying processes on the chemical and physical properties of safflower: Towards a multidimensional quality evaluation model

**DOI:** 10.1371/journal.pone.0339180

**Published:** 2026-01-02

**Authors:** Juan Li, Rui Wang, Cuiping Chen, Xulong Huang, Bin Xian, Tang Lv, Chaoxiang Ren, Qinghua Wu, Feiyan Wen, Jie Yan, Jin Pei

**Affiliations:** 1 Chinese Medicine Germplasm Resources Innovation and Effective Uses Key Laboratory of Sichuan Province, Chengdu University of Traditional Chinese Medicine, Chengdu, China; 2 College of Pharmacy, Chengdu University of Traditional Chinese Medicine, Chengdu, China; 3 Key Laboratory of Standardization of Chinese Medicine (Chengdu University of Traditional Chinese Medicine), Ministry of Education, Chengdu University of Traditional Chinese Medicine, Chengdu, China; University of Delhi, INDIA

## Abstract

The drying process is a critical step in determining the quality of safflower (*Carthamus tinctorius* L.). This study aims to systematically evaluate the effects of different drying methods on the chemical composition, color, morphology, odor, and microstructure of safflower dried petals, and to establish a comprehensive quality evaluation model for safflower based on machine learning. The results showed that drying methods significantly altered the chemical composition of safflower. Freeze-dried samples exhibited significantly higher levels of the active components hydroxysafflower yellow pigment A and anhydrosafflower yellow pigment B compared to other methods (*p* < 0.05), presenting a bright orange color and a mild odor. Microscopic structure and morphological analysis indicate that freeze-dried safflower effectively preserves its morphological characteristics, with a clear arrangement of cells and lower overall shrinkage. Based on nineteen quality parameters, nine quality evaluation models for safflower were constructed. The multiclassification decision forest model achieved a prediction accuracy of 89.1%. The importance analysis of quality parameters revealed that the B, G, and R color features in the RGB color mode are the most critical indicators for evaluating safflower quality. This study provides key basis for optimizing the drying process of safflower. The comprehensive evaluation model established provides a technical foundation for intelligent evaluation and standardized control of safflower quality, which is of significant practical value for improving safflower quality and promoting the standardized development of the industry.

## Introduction

Safflower (*Carthamus tinctorius* L.) is a multifunctional economic crop. It is widely cultivated in more than 60 countries or regions in the world [1,2]. The florets, seeds, stems and leaves can be utilized for food, dye, medicine, feed and health care food [3,4]. In 1993, the Food and Agriculture Organization of the United Nations (FAO) formally listed safflower as a dual-use plant for medicinal and food purposes [5]. The petals contain yellow and red pigments which are commonly used for color mixing in food, clothing and cosmetics [6]. It is also made into safflower wine, safflower tea and safflower medicinal dishes with health benefits [7,8]. *Carthami Flos* is the dried petals of safflower and contains active constituents such as flavonoids. Its effects on the system of cardiovascular, immune, reproductive, nervous and digestive systems have been reported [9].

Fresh safflower has a high moisture content and is prone to spoilage during storage and transportation resulting in waste of resources. Therefore, the drying process is the most important post-harvest step for safflower storage and transportation as well as quality formation. Factors like temperature, pressure and drying medium associated with this process affect the physicochemical properties and biological activity of the dried product to varying degrees, resulting in differences in quality due to morphological shrinkage, color browning, and loss of active ingredients [10–13]. The traditional methods of processing safflower at its origin are mainly shade-drying and sun-drying, which do not involve external heat sources [14,15]. Prolonged dry times caused by climate change may lead to microbial growth and reproduction, while simultaneously causing the loss of bioactive compounds [16]. In response to these problems, various industrial dryers have been designed and developed in recent years [17]. Oven drying with hot air is considered a low-cost, time-efficient, and highly controllable drying method. It utilizes circulating hot air to evaporate and remove moisture from materials to achieve drying. However, high temperatures increase the likelihood of degradation of bioactive compounds and deterioration in product visual quality [18]. Decompression drying and freeze-drying in vacuum reduce air pressure by vacuuming in closed containers, thus realizing accelerated loss of moisture at lower temperatures. These methods effectively avoid decomposition or deterioration of the bioactive compounds of the product due to high temperatures. The reduced exposure of materials to air during the drying process prevents sample oxidation [19]. In addition, freeze-drying makes the moisture in the material directly removed by sublimation from the solid state into the gaseous state, which can effectively maintain the structure and color of the material [20]. Prior studies have demonstrated that vacuum freeze-drying and microwave drying are superior to air-drying, sun-drying, and oven-drying in terms of retaining the primary active components of safflower (such as hydroxysafflor yellow A and safflor yellow A). Additionally, safflower processed by vacuum freeze-drying exhibits better appearance than that dried by microwave. Nevertheless, it also suffers from issues such as high energy consumption, high operating costs, and lengthy processing times [21].

Drying process, as a key step in safflower processing, directly affects the retention rate of safflower active ingredients and appearance quality. The current Chinese Pharmacopoeia stipulates the conventional physical and chemical indexes of safflower. In actual production, the evaluation of safflower quality still mainly relies on sensory evaluation or quantitative descriptive analysis [22]. While studies have explored the effects of different drying methods on the appearance and chemical composition of safflower, there is currently a lack of work incorporating drying parameters into the safflower quality evaluation system. Machine learning methods can integrate key parameters to build models, reasonably predict product quality, and obtain the conditions required for high-quality products. Yoon et al. [23] showed that machine learning methods can effectively monitor cannabinoid composition and determine optimal drying endpoints to predict changes in cannabis quality metrics during hot-air and cold-air drying. Przybył et al. [24] developed an artificial neural network based on variables represented by 46 image descriptors for predicting the quality category of rhubarb juice powder during spray drying. In another study, Huang et al. [25] used Support Vector Machine to predict the degree and type of roast of oven-roasted coffee beans. However, there are currently no studies that have established a model for evaluating the quality of safflower using machine learning algorithms based on different drying methods.

Therefore, the study collected fresh safflower samples (F) and processed them by natural shade drying (NSD), double fifteen shade drying (DFSD), sun drying (SD), freeze drying (FD), decompression drying (DD), and oven drying at 40^∘^C, 60^∘^C, and 80^∘^C (OD40, OD60, and OD80). Changes in chemical constituents in safflower before and after drying were analyzed by Ultra performance liquid chromatography-quadrupole-electrostatic field orbit trap high-resolution mass spectrometry (UPLC-Q-Orbitrap MS), Gas chromatography-mass spectrometry (GC-MS) and High performance liquid chromatography (HPLC). The effects of different drying methods on the macroscopic characters and microstructure of safflower were analyzed by sensory evaluation combined with spectrophotometer and scanning electron microscopy (SEM). The purpose of this paper is to explore the effects of eight drying methods on the chemical composition, macroscopic characteristics, and microstructure of safflower, and to incorporate drying methods into the safflower quality evaluation system to construct a safflower quality evaluation model. This will establish a more comprehensive and objective safflower quality evaluation method and guide producers in adopting appropriate drying methods to produce high-quality products.

## Materials and methods

### Materials

The fresh safflower used in the experiment was Sichuan safflower harvested from the Sichuan Jianyang Safflower Base (Jianyang, China). Sampling was conducted during the phenological period (April to May), and the safflower was picked during its peak flowering period when it turned from yellow to red. The safflower samples used to construct the machine learning model dataset comprised laboratory-made dried safflower, field-collected dried safflower, and commercially purchased dried safflower. These samples were sourced from Sichuan, Henan, Xinjiang, Yunnan, and Gansu provinces in China, with each sample featuring three biological replicates for subsequent modeling analysis. Information on the safflower samples used in the experiment is provided in Supporting information S1 Table.

### Drying methods

In this study, eight drying methods were used to investigate the drying quality of safflower. The drying methods are shown in Table 1. Information of the equipment used for drying is shown in Supporting information S1 Fig. According to the Pharmacopoeia of the People’s Republic of China 2020, the moisture content of dried safflower should not exceed 13% [26].

**Table 1:** Different drying methods for fresh safflower.

Drying method	Specific operations and conditions
*NSD*	The samples were dried in a well-ventilated room, protected from light, and turned once per hour (temperature 20 ^∘^C, humidity 54%)
*DFSD*	The samples were dried in a “double fifteen” drying room, turning once per hour (temperature 15 ^∘^C, humidity 15%)
*SD*	In sunny weather, the samples were spread out in a sunny place to dry, and turned once per hour (temperature 28 ^∘^C, humidity 43%)
*FD*	Drying with vacuum freeze dryers
*DD*	Drying at 30 ^∘^C in a depressurized oven
*OD*	Continuous drying in an electric blast oven (at 40 ^∘^C, 60 ^∘^C and 80 ^∘^C)

Note: The drying endpoint is defined as a moisture content of less than 13%. Moisture content measurements are performed in parallel three times for each drying method. Safflower is spread out to a thickness of 0.5 to 1 cm. The drying oven is preheated for 30 minutes before use to stabilise the temperature.

### UPLC-Q-Orbitrap MS analysis

The fresh safflower samples frozen in liquid nitrogen were ground, and the safflower samples with different drying treatments were powdered and sieved. For each dry sample, 50 mg of powder was weighed into a 2 ml sterilized centrifuge tube and dissolved in 1.0 ml of 70% aqueous methanol. The samples were placed in a refrigerator at 4 ^∘^C overnight, during which they were vortexed 3 times and then centrifuged at 1 × 104 g for 10 min. The supernatant was taken and filtered through a microporous filter membrane (0.22 μm) to obtain the filtrate, which was stored in the inlet bottle. Three replicates were set up for both fresh samples and each drying method.

The qualitative analysis of compounds differing in different drying methods was performed by UPLC-Q-Orbitrap MS (Thermo Fisher Scientific, USA) technique with chromatographic conditions referring to Wang et al. [27]. For mass spectrometry, an electrospray ionization source was used in positive and negative ionization mode with a positive spray voltage of 2.50 kV, a negative spray voltage of 2.50 kV, a sheath gas of 20 arb, and an auxiliary gas of 10 arb. The capillary temperature was 225 ^∘^C, and the full scan was performed at a resolution of 70,000, with a mass scanning range of 50 to 1,500 m/z, and the collision voltage was 20 eV. Unnecessary MS/MS information was removed using dynamic exclusion.

The raw data were imported into the Compound Discoverer 2.0 software for comparison and peak extraction, and the molecular formula was fitted. The measured secondary fragment spectra were matched with the mzCloud and mzVault databases to analyze and identify compounds. The ion chromatographic peak area data of the qualitatively identified compounds were imported into SIMCA 14.1 software. After normalization, the metabolites were analyzed Partial Least Squares-Discriminant Analysis (PLS-DA), and then screened for differences in metabolites before and after drying by using Variable Importance in Project (VIP) in combination with fold change of individual compounds.

### GC-MS analysis

The fresh samples and samples dried by different drying methods were transferred into 15 ml extraction vials and sealed quickly. The SPME extraction fiber head was aged at 250 ^∘^C at the GC-MS (Agilent Technologies, USA) inlet to no impurity peak. The sample bottles were placed on a solid-phase microextraction device with the temperature set to 80 ^∘^C. The bottle is then placed in the extraction unit and preheated for 15 min. The SPME extraction head was inserted into the headspace part of the sample through the cap, and the fiber head was pushed out. The extraction head was about 1.0 cm higher than the upper surface of the sample, and the headspace extraction was performed for 60 min. The fiber head was withdrawn and the extraction head was pulled out from the sample bottles. Then the extraction head was inserted into the GC-MS injection port to push out the fiber head, which was desorbed at 260 ^∘^C for 1 min and injected for analysis.

A column of DB-17 MS (20.0 m × 250 μm, 0.25 μm) was used for chromatographic separation. The mass spectrometry was performed using an EI source, an ionization source with an electron energy of 70 eV, an ion source temperature of 220 ^∘^C, a quadrupole temperature of 150 ^∘^C, and a mass range of 20 to 550 u. The qualitative analyses were carried out using the mass spectrometry database, NIST11, the retention times and retention indices of the detected components. The relative content data of qualitatively identified compounds were imported into SIMCA 14.1 software for PLS-DA analysis to screen for differential metabolites before and after drying with VIP values. The percentage of the peak area of the identified components to the sum of the areas of all identified components was used as the quantitative result. The formula was calculated as follows [28]:

$$Ci=\frac{Ai}{A1+A2+\cdots +Ai+\cdots +An}\times 100\%$$ (1)

Where Ci is the content of an identified component (%); Ai is the peak area of an identified component; n is the total number of identified components.

### UPLC multi-component quantitative analysis

Take the appropriate amount of each control, dilute 2, 4, 8, 16, 32 times with methanol by 2-fold dilution method to obtain the mixed standard solution of each concentration and prepare the standard curve. Take 0.4 g of sample powder, put it into a stoppered conical flask, add 50 ml of purified water, weigh it accurately, ultrasonic treatment for 40 minutes, cool it down, weigh it again, and make up the loss of weight with purified water, shake it well, filter it, and take the filtrate, obtain the test material.

For the chromatographic conditions, the column was an Agilent ZORBAX SB-C18 RRHT UPLC column (100 mm × 4.6 mm, 1.8 μm), and the mobile phase was acetonitrile (A)-0.1% formic acid aqueous solution (B), and the elution gradient was as follows: 0–5 min, 2–5% A; 5–10 min, 5%–10% A; 10–30 min, 10%–20% A; 30–45 min, 20% A; 45–46 min, 20–2% A; 46–55 min, 2% A. The detection wavelength was 360 nm; the volume flow rate was 0.2 ml/min; the column temperature was 30 ^∘^C; the injection volume was 2 μl.

To assess the precision of the method, the sample solution was injected six times consecutively, using HSYA as the reference peak based on peak stability, high response values, and large peak areas. The sample solution was injected and detected at 0, 4, 8, 12, 16, 24, and 48 hours to assess the stability of the method. Six parallel samples of the sample solution were prepared and injected for detection to assess the repeatability of the method. Take 6 samples of NSD dried safflower, place them in 10 ml volumetric flasks, and add eight reference standards at 100% of the known content to each sample to conduct recovery experiments to further ensure the accuracy of the method.

### Color, odor, and morphological properties analysis

The overall and localized traits of safflower samples obtained from different drying treatments were photographed with a single-lens reflex camera (SLR) and a stereo microscope. The safflower samples were observed in terms of color, morphology, odor and other traits. The L*, a* and b* values of safflower powder before and after drying were determined using a spectrophotometer. The total color difference (ΔE*, Eq 2) and the browning index (BI, Eqs 3 and 4) were calculated from the measured L*, a* and b* values using the following formulas [29].

$$\Delta E^{*}=\frac{\left[(\Delta L^{*}{)}^{2}+(\Delta a^{*}{)}^{2}+(\Delta b^{*}{)}^{2}\right]}{2}$$ (2)

$$BI=\frac{\left[100(X-0.31)\right]}{0.17}$$ (3)

$$X=\frac{a^{*}+1.75L^{*}}{5.645L^{*}+a^{*}-0.012b^{*}}$$ (4)

### SEM analysis

The safflower samples were fixed on a sample stage and sprayed with gold 6 times for 60 seconds each time. The shrinkage of the corolla tube and inner and outer surface of corolla of safflower samples after different drying treatments were observed by SEM.

### Machine learning model construction for safflower quality evaluation

The sensory characteristics of safflower such as color, texture, aroma, and morphology serve as key criteria for quality grading. Manual sensory evaluation remains the primary method for assessing safflower quality. From July 20 to July 22, 2023, this study assembled an evaluation panel comprising four experts specializing in Chinese medicinal resources and identification. The study received written informed consent from the four experts. Experts conducted sensory evaluations and quality grading of safflower according to the document (84) No. 72, Annex, “Standards for the Commodity Specification of 76 Herbal Medicines” – Safflower [30]. Each sample was evaluated in parallel three times, and the grading criteria are shown in Table 2. All samples were randomly assigned three-digit codes and presented to experts in random order for blind evaluation. Samples adhered to uniform standards, with weighed (5.00 ± 0.01) g safflower specimens placed in transparent, lidless disposable plastic petri dishes (90mm diameter, 15mm height) for sensory assessment. Each expert independently evaluated all samples, taking a 10-minute break after every 5 samples to mitigate sensory fatigue. Each sample was evaluated three times. After all experts completed independent evaluations, the research team aggregated each expert’s scores. Any sample receiving inconsistent classifications among experts was excluded.

**Table 2:** Sensory evaluation standard of safflower class classification.

Class	Evaluation Standard
*Class*1	Dried. Tubular flowers crumpled and bent, in clusters or scattered. Surface deep red, bright red, slightly yellowish. Soft texture, aroma, taste slightly bitter, without branches and leaves, impurities, insect moths, and mold.
*Class*2	Dried. Tubular flowers crumpled and bent, in clusters or scattered. Surface light red, dark red or yellow. Soft texture, aroma, and slightly bitter taste, without branches and leaves, impurities, insect moths, and mold.
*Class*3	Dried. Tubular flowers crumpled and bent, in clusters or scattered. Surface dark reddish black or more yellow. Soft texture, aroma, and slightly bitter taste. Leaves and branches not more than 5%, no impurities, moths, and mold.
*Unqualified*	Dried. Tubular flowers crumpled and bent, in clusters or scattered. Surface dark red and black or yellow more. Crisp texture, light aroma, slightly bitter taste, branches and leaves not more than 5%, with impurities, moth, and mold.

Machine learning is used to construct predictive models for the quality of dried safflower. On the Azure Machine Learning (AML, https://studio.azureml.net/) platform, the following models are used for the prediction task: two-classification average perceptron, two-classification Bayesian point machine, two-classification decision forest, two-classification local deep support vector machine, two-classification logistic regression, two-classification neural network, two-classification support vector machine, multiclassification decision forest, and multiclassification neural network. Logistic regression is one of the most fundamental models in statistics and machine learning, offering strong interpretability and probabilistic outputs. The research uses two-classification logistic regression model as a benchmark. The perceptron is a simple linear classification algorithm that attempts to find a hyperplane separating two classes of data by directly adjusting weights. The “average” technique enhances model generalization by averaging the weight vectors across all iterations during training, thereby reducing overfitting. Decision forests build multiple decision trees, each trained on a random subset of the training data and feature subset. This approach resists overfitting and demonstrates excellent accuracy on tabular data [31]. Bayesian point machines are linear classifiers based on Bayesian theory. Their Bayesian nature makes them resistant to overfitting and particularly robust with limited data [32]. Support vector machine utilizes kernel functions to map low-dimensional feature data into high-dimensional space, where it seeks the maximum margin hyperplane to minimize the distance between samples and the hyperplane. This approach is well-suited for analyzing small-sample, non-linear, and high-dimensional data [33]. Neural networks are powerful general-purpose function approximators capable of capturing complex nonlinear relationships and interaction effects in data [34,35]. It was possible to verify whether our problem contained hidden patterns requiring deep architectures to learn.

During our collection of safflower samples, we observed an imbalance in sample categories. This aligns with the actual distribution ratio of various safflower products in the market, but such data imbalance significantly impacts model training effectiveness. To mitigate this issue, k repetitions were performed on minority samples in the training dataset (k = 3). This involves repeating measurements on minority samples k times to double the minority data in the augmented training dataset. Data imbalance is a common phenomenon [36], and data augmentation using repeated measurements has been employed as a countermeasure [37]. Augmentation methods based on parallel measurement data preserve the inherent variability of the samples. Related approaches have been proven effective in enhancing model sensitivity in blood glucose classification studies [38].

Each sample collected for the machine learning dataset has 1 category label and 19 feature attribute categories. The category label represents the sensory evaluation quality grade of safflower. The 19 feature attributes respectively record each safflower sample’s drying method, drying temperature, color values measured by spectrophotometer (L*, a*, b*, ΔE*), color values calculated based on RGB (R, G, B) and brightness, storage duration, as well as the content of 8 chemical components including HSYA, AHSYB, HKT, KD, HKR, HKD, HAG, and KR. All continuous numerical features underwent Z-score normalization. Safflower quality grades and different drying methods were quantified: safflower quality was defined as 1 for Grade 1, 2 for Grade 2, 3 for Grade 3, and 4 for unqualified. NSD was defined as 1, DFSD was 2, SD was 3, FD was 4, DD was 5, and OD was 6.

Model hyperparameters were not manually tuned, but instead we employed AML’s built-in modules for automated optimization. Model performance was evaluated using metrics including accuracy, precision, recall, and F1-score. The entire dataset was randomly split into training and independent test sets at ratios of 7:3, 8:2, and 8.5:1.5. All models were trained exclusively on the training set.

### Statistical analysis

The differences between different treatment groups were compared using one-way analysis of variance (ANOVA). The SPSS 26.0 software was used for analysis, and Duncan’s multiple range test was applied. The SIMCA 14.1 software is used for performing PLS-DA and VIP analysis. The results of the correlation analysis between drying temperature (DTEM), drying time (DT), moisture content (MC) of dried safflower, main active ingredients, and color values were obtained using OriginPro 2021 software and expressed as Pearson correlation coefficients. The significance level was set at *p* < 0.05 or *p* < 0.01. Data are expressed as mean ± standard deviation (SD).

## Results

### Identification and analysis of metabolites in eight kinds of dried safflower

A total of 155 components were identified in all the samples, most of which were primary metabolites such as organic acids and amino acids involved in the basic plant metabolism, including 34 amino acids and peptides, 29 organic acids, 23 lipids, 11 carbohydrates and derivatives, 12 nucleosides, nucleotides and derivatives, 2 vitamins, and a few secondary metabolites including 14 flavonoids, 5 alkaloids, 5 amines, 3 terpenoids, 2 aldehydes and 15 other constituents. The details are shown in S2 Table.

**Fig 1:**
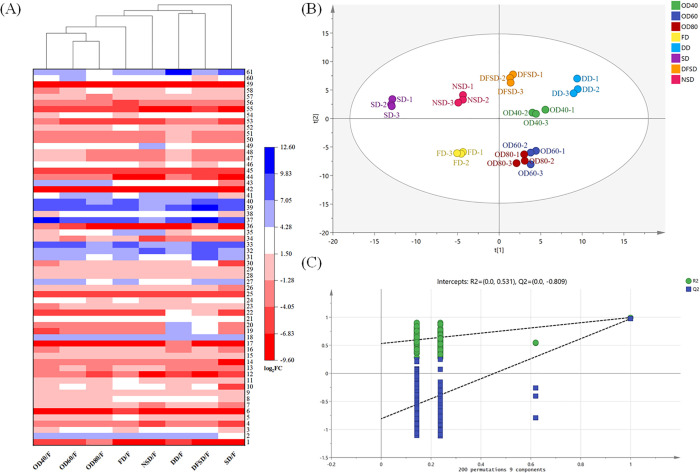
UPLC-Q-Orbitrap MS analysis of different drying methods. (A) Cluster heat map of compounds that differ between fresh samples and different drying methods; (B) PLS-DA score scatter plot; (C) PLS-DA permutation test diagram.

The compounds in the samples of different drying methods were all the same kind, but the relative contents were different. As a result, 61 compounds with differences before and after the drying of safflower were identified by using |log_2_FC| ≥ 1 and VIP ≥ 1 as the screening indexes by analyzing the ion chromatographic peak area data. The results are shown in Fig 1(A) and Supporting information S3 Table. Among the 61 compounds, most of them showed the same increasing and decreasing trend in different drying methods. Compared with the fresh samples, the composition of components with increased and decreased levels is complex. The constituents showing significant reduction include various organic acids, vitamins, glycosides, terpenoids, and lipids and other components such as D-saccharic acid, D-(-)-quinic acid, pantothenic acid, chlorogenic acid, D-glucopyranoside, trifolin, ligustilide, nootkatone, pentadecanoic acid, and linoleoyl ethanolamide. The components significantly increased were amino acids, carbohydrates, flavonoids, phenolic acids and other components such as L-glutamic acid, 4-guanidinobutyric acid, 3-[(carboxycarbonyl)amino]-L-alanine, galacturonic acid, δ-gluconic acid δ-lactone, quercetin-3β-D-glucoside, quercetin, genistein, caffeic acid.

Flavonoids are the main active components of safflower [39]. The HSYA and kaempferol are safflower flavonoids. Their content is an important index to evaluate the quality of safflower. The log_2_FC values of HSYA for the eight drying methods compared with fresh samples were –1.10 (NSD), –0.87 (DFSD), –1.04 (SD), –0.31 (FD), –0.84 (DD), –0.82 (OD40), –0.57 (OD60) and –0.68 (OD80), respectively. The log2FC values of kaempferol for the eight drying methods compared with fresh samples were –7.96 (NSD), –8.00 (DFSD), –8.45 (SD), –8.39 (FD), –7.56 (DD), –7.93 (OD40), –7.58 (OD60) and –7.90 (OD80), respectively.

The results of PLS-DA analysis are shown in Fig 1(B), and the spatial distribution of the samples dried by different drying methods has specific regions. The distribution of the points in each group was concentrated, and the intra-group differences were small. The NSD and SD samples were located in the same quadrant, indicating the similarity of the chemical composition of safflower dried by the traditional drying method. The OD60 and OD80 samples were located in the same 4th quadrant and partially overlapped, suggesting that the difference in chemical composition between the two drying methods was minimal. Only the FD sample was located in the 3rd quadrant, indicating that the chemical composition of the FD sample was more different from the other drying methods. The analysis of the PLS-DA model with 200 permutation test (Fig 1C) showed that there was no overfitting in the model.

### Identification and analysis of volatile components in eight kinds of dried safflower

The main aroma components of safflower are aldehydes, ketones, esters, alcohols and alkenes [40]. The fresh safflower and safflower samples treated with different drying methods identified a total of 76 volatile components, mainly hydrocarbons, terpenes, alcohols, aldehydes, esters, ethers and ketones (see Supporting information S4 Table). Based on the comparative screening (match ≥ 80) of database NIST11, there were 12, 14, 25, 24, 22, 26, 29, 32 and 35 compounds identified from F, NSD, DFSD, SD, FD, DD, OD40, OD60 and OD80 samples, with a relative content of 41.07%, 70.75%, 77.95%, 81.23%, 73.10%, 61.77%, 55.29%, 56.35% and 55.49% of the total volatile components of the group. The components detected only in the fresh samples were dimethyl phthalate, myrtenol, trans-beta-ionone (relative content >1%). Components detected only in dried samples were eucarvone, 3-ethyl-3-methylheptane, tetradecane, cyperene, 9,10-dehydro-isolongifolene, humulene, 1-pentadecene, 1-tridecene alpha-bulnesene, (+/-)-dihydroactinidiolide, caryophyllene oxide, alpha-caryophylladienol, 4-allyltoluene, hexahydrofarnesylacetone, supraene (relative content >1%).

**Fig 2:**
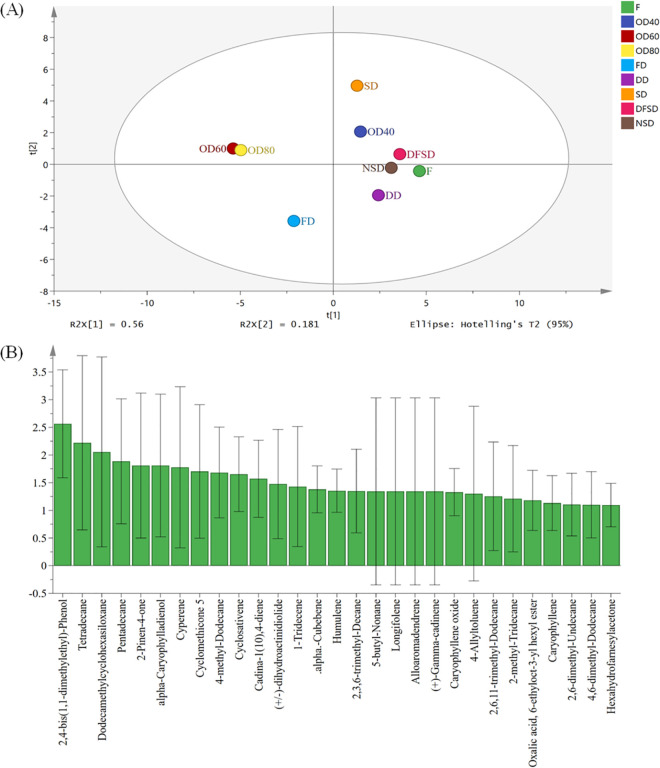
GC-MS analysis of different drying methods. (A) PLS-DA score scatter plot; (B) PLS-DA VIP value diagram.

To investigate the differences in the volatile components of safflower dried by different drying methods, the relative content data of the components determined by GC-MS were imported into SIMCA 14.1 software for PLS-DA analysis. The results (Fig 2A) showed that fresh samples of safflower and samples processed by different drying methods could be effectively separated. The highest similarity of volatile components was observed in the OD60 and OD80 samples, while the FD sample showed the greatest difference from the other samples. There were 29 compounds (Fig 2B) with VIP values ≥ 1, and these compounds played an important role in the classification of samples from different drying methods using the PLS-DA method.

### Quantification of active ingredients in eight kinds of dried safflower

**Fig 3:**
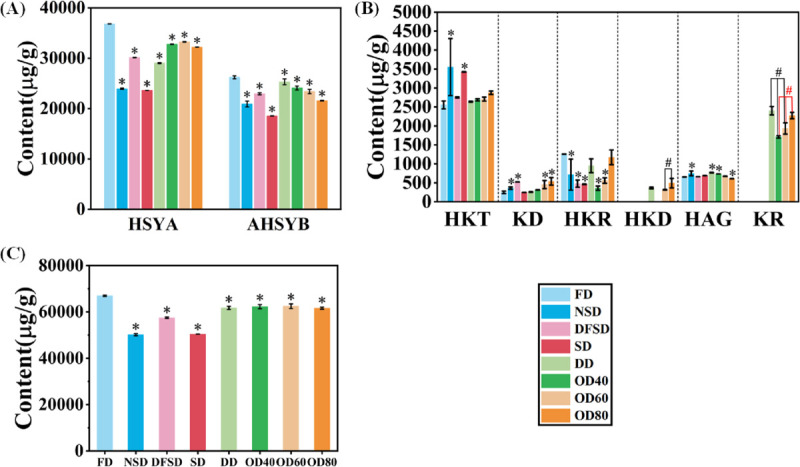
The contents of chalcone component (A), kaempferol glycoside component (B) and the total content of the two components (C) in safflower samples dried by different drying methods. HSYA is Hydroxysafflor yellow A; AHSYB is Anhydrosafflor yellow B; HKT is 6-Hydroxykaempferol-3,6,7- tri-O-glucoside; HKR is 6-Hydroxykaempferol-3-O-rutoside-6-O-glucoside; HKD is 6-Hydroxykaempferol-3,6-di-O-glucoside; HAG is 6-Hydroxyapigenin-6-O-glucoside- 7-O-glucuronic acid; KR is Kaempferol-3-O-rutoside; KD is Kaempferol-3,7-di-O- glucoside.“*” indicates a significant difference in data compared to FD samples (*p* < 0.05). “#” indicates a significant difference in the comparison between the groups. Values are mean ± SD (n=3).

The relative standard deviations (RSD) of the retention times of the common peaks in the precision, stability, and repeatability tests (n = 6) were 1.74%, 2.25%, and 2.54%, respectively. The RSD (n = 6) for peak area was 1.18%, 2.61%, and 2.37%, respectively. This indicates that the precision, stability, and repeatability of the analytical method are favorable. The recovery rates for the eight reference standards ranged from 98.1% to 99.2%, with RSD (n=6) values of 0.75% to 2.01%, indicating that the accuracy of the analytical method is favorable. The UPLC liquid chromatograms of the mixed standards are shown in Supporting information S2 Fig. The eight flavonoids could be well separated. Standard curves, linearity ranges, and correlation coefficients (r^2^) for each component are presented in Supporting information S5 Table. The content of each index component was calculated based on the peak area. The results are shown in Supporting information S6 Table and Fig 3. The contents of HSYA and AHSYB were significantly higher in the FD samples than in the other drying methods, and the total content of the active ingredients was the highest at 66931.13 ± 290.20 ug/g (*p*<0.05). HSYA content: OD > NSD > SD, consistent with the results of Zhang et al. [41]. The NSD and SD samples had significantly higher HKT content than the other drying methods, but the lowest total active ingredient content was 50200.40 ± 436.86 ug/g and 50450.68 ± 25.51 ug/g, respectively (*p*<0.05). The total content of active ingredients in safflower samples obtained by the traditional NSD and SD methods was lower than that of the FD, DD and OD methods. The OD60 and OD40 samples had the second-highest content of active components after that of the FD samples. The two components, HKD and KR, were not detected in the FD, NSD, DFSD and SD samples, but could be detected in the DD and OD samples.

### Impacts of eight drying methods on the color, odor, and morphological properties of safflower

**Fig 4:**
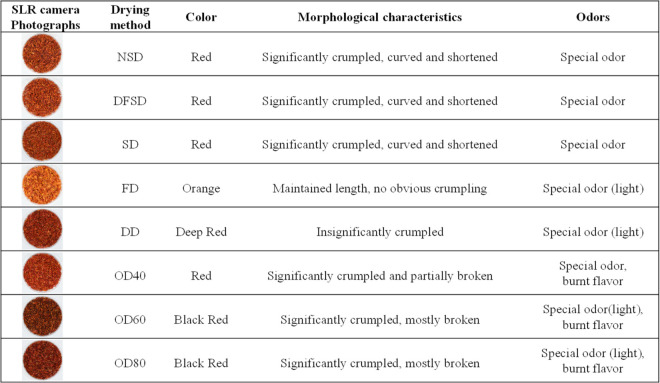
Characters of safflowers obtained by different drying methods.

**Fig 5:**
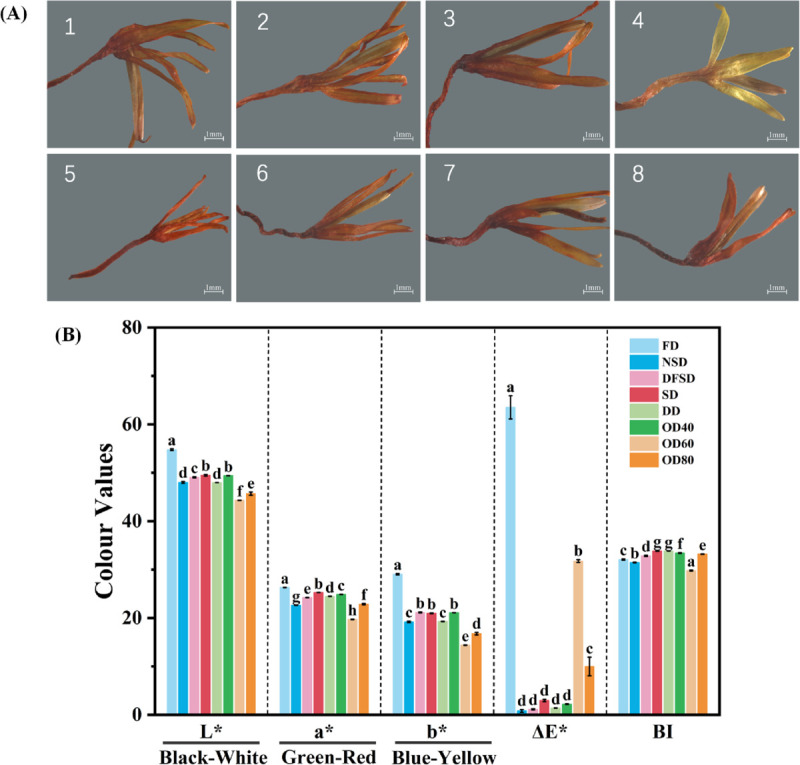
Microscopic observation of morphology and quantitative color analysis of safflower obtained by different drying methods. (A) Safflower samples photographed by stereo microscope. 1-NSD; 2-DFSD; 3-SD; 4-FD; 5-DD; 6-OD40; 7-OD60; 8-OD80; (B) Color values of samples with different drying methods. Different lowercase letters indicate significant differences at *p* < 0.05. Values are mean ± SD (n=3).

The study observed and described the color, morphology and odor of safflower samples obtained through different drying processes as shown in Fig 4. The photographs of safflower taken with a stereo microscope are shown in Fig 5(A).

The color of dried safflower corolla was reddish-yellow crosswise and the corolla tube was darker. NSD, DD and FD samples dried at lower temperatures were less wrinkled and curved. The FD samples were the closest in morphology to the fresh samples, with the least shrinkage and the lightest odor. The OD samples at the three temperatures were more wrinkled and bent, and the safflower obtained was darker in color. Moreover, the bending of safflower would increase with increasing temperature, the texture would become dry and fragile, and the special flavor would fade to burnt flavor.

The color parameters of safflower dried by different drying methods are shown in Fig 5(B). The highest L* (54.76 ± 0.18), a* (26.31 ± 0.03) and b* (29.04 ± 0.16) values were observed for the FD samples as compared to the other drying methods. This agrees with the findings of Geng et al. that the highest values of L*, a* and b* were found in the FD samples [42]. In addition, SD (49.49 ± 0.17) and OD40 (49.40 ± 0.04) samples showed higher levels of L* values than the other samples in the following order: SD > OD40 > DFSD > NSD > DD > OD80 > OD60. Safflower is rich in flavonoids such as safflower yellow pigment and safflower red pigment. The yellow and red pigments in safflower are quite unstable. The red pigment is unstable to heat, while the yellow pigment is sensitive to light [43–45]. The color change during drying could be a result of this. All the dried samples had different degrees of ΔE*. The FD samples had the highest ΔE* (63.53 ± 2.39), followed by OD60 (31.75 ± 0.31). The BI is a quantitative measure of the degree of color change of products during storage and processing due to oxidation, enzymatic reactions and heat treatment, etc. The lowest value of BI was found in OD60 (29.79 ± 0.10), followed by NSD (31.45 ± 0.08), FD (32.06 ± 0.12), DFSD (32.83 ± 0.12), OD80 (33.21 ± 0.06), and OD40 (33.41 ± 0.07). SD (33.85 ± 0.10) and DD (33.84 ± 0.02) samples had the highest BI values.

### Impacts of eight drying methods on the microstructure of safflower

**Fig 6:**
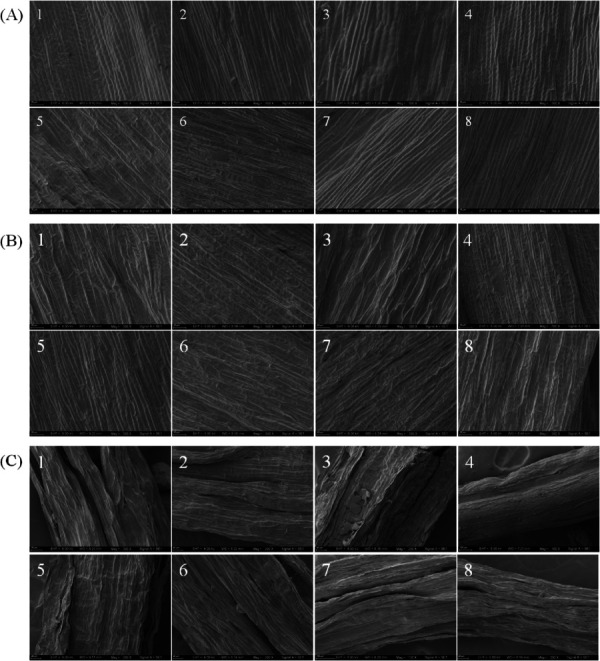
SEM images of the surface structure of safflower with different drying methods. (A) Corolla (inner surface); (B) Corolla (outer surface); (C) Corolla tube. 1-NSD; 2-DFSD; 3-SD; 4-FD; 5-DD; 6-OD40; 7-OD60; 8-OD80.

The results of the crumpling of corolla and corolla tube of safflower samples after different drying treatments were photographed using SEM as shown in Fig 6. The inner and outer surfaces of dried safflower corolla were different (Fig 6A and B). The outer surface of the NSD, DFSD, SD, FD, OD60 and OD80 samples was wrinkled and concave, with poorly aligned cells and an overall skewed and twisted appearance, whereas the inner surface was full and convex, with neatly aligned cells in an elongated shape. The DD sample was in the opposite state, with the outer surface being full and the inner surface concave. The OD40 samples are concave on both the inner and outer surfaces. The crumpling degree of the corolla tube part of safflower after drying was large (Fig 6C). Except for the FD sample which had a smooth surface and neat cell arrangement, the rest of the samples had a large crumpling of the corolla tube.

### Correlation analysis between safflower drying parameters and quality indicators

**Fig 7:**
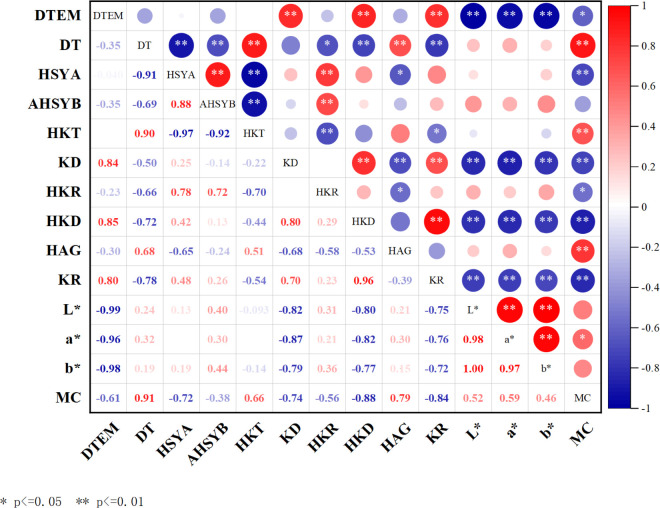
Pearson correlation analysis of safflower drying parameters and quality indicators. “*” indicates significant correlation at the *p* ≤ 0.05 level (two-tailed); “**” indicates significant correlation at the *p* ≤ 0.01 level (two-tailed).

This study systematically investigated the effects of DTEM, DT, and MC on the content of major active components and color indices of safflower through Pearson correlation analysis, with the results shown in Fig 7. The analysis revealed that DTEM was negatively correlated with the content of HSYA and AHSYB. Higher DTEM led to the degradation of these two major active components, with a more pronounced negative impact on AHSYB. DT showed a significant negative correlation with these two components (*p* ≤ 0.01). Prolonging DT is detrimental to the retention of HSYA and AHSYB. Most kaempferol glycoside components (KD, HKD, KR) exhibited a significant positive correlation with DTEM (correlation coefficients ranging from 0.80 to 0.85, *p* ≤ 0.01). However, DT showed a negative correlation with these components. It is worth noting that HKT and HAG content showed a strong positive correlation with DT (*p* ≤ 0.01). Color is an important indicator for evaluating the appearance grade of safflower. DTEM showed a significant negative correlation with L*, a*, and b* values (correlation coefficients ranging from –0.96 to 0-0.99, *p* ≤ 0.01). This indicates that as DTEM increases, the brightness of safflower significantly decreases, red saturation decreases, yellow fades, and quality deteriorates. The main active components HSYA and AHSYB showed a positive correlation with color values L*, a*, and b*. KD, HKD, and KR showed significant negative correlations with brightness L*, a*, and b* values (correlation coefficients ranging from -0.75 to -0.82, *p* ≤ 0.01), indicating that the retention of active components is synchronized with the formation of color appearance. Additionally, the correlation patterns between final MC and various parameters were highly consistent with DT.

### Machine learning modeling safflower quality comprehensive

**Fig 8:**
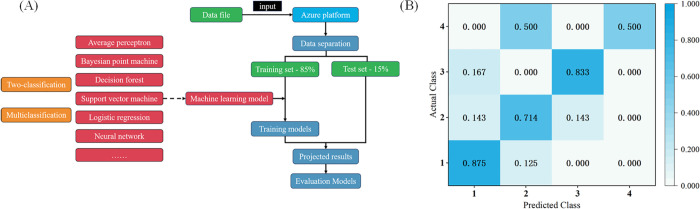
The construction of a machine learning model for safflower quality evaluation. (A) Machine learning flowchart; (B)The confusion matrix of multiclassification decision forest.

A total of 144 samples were collected for building machine learning models, and the sample information is shown in Supporting information S7 Table. The machine learning flowchart is shown in Fig 8(A). The accuracy and precision of various machine learning models are improved with a training-to-test ratio of 8.5:1.5. Several machine learning models were comprehensively employed in this study to evaluate safflower quality, with Table 3 summarizing the predictive performance metrics of each model. The two-classification decision forest performs excellently on accuracy (0.826) and precision (0.929), with about 8.7% and 21.5% improvements over the baseline respectively. The two-classification local deep support vector machine shows enhanced recall (0.933) and F1 score (0.875), demonstrating more balanced performance. Among models with relatively lower performance, the two-classification support vector machine significantly underperformed the baseline in accuracy (0.630) and precision (0.630). The multiclassification neural network showed suboptimal recall (0.627) and F1 score (0.653). The two-classification Bayesian point machine failed to surpass the baseline across most metrics. It is worth noting that the multiclassification decision forest achieved the highest accuracy (0.891) among all models, representing a 15.2% improvement over the baseline and demonstrating its advantage in complex classification tasks. However, its confusion matrix (Fig 8B) reveals disparities in the model’s recognition capabilities across categories: class 1 and class 3 achieved high recognition rates (87.5% and 83.3%, respectively), while class 2 and class 4 showed poorer recognition performance. Notably, only 50% of class 4 samples were correctly classified, with the remainder misclassified as class 2. This indicates confusion between certain categories, potentially due to feature overlap.

**Table 3:** Prediction evaluation indicators of each model.

Model	Accuracy	Precision	Recall	F1
Two−classification logistic regression	0.739	0.714	1.000	0.833
Two−classification average perceptron	0.783	0.857	0.800	0.828
Two−classification Bayesian point machine	0.696	0.750	0.800	0.774
Two−classification decision forest	0.826	0.929	0.813	0.867
Two−classification localized deep support vector machine	0.826	0.824	0.933	0.875
Two−classification neural network	0.826	0.867	0.867	0.867
Two−classification support vector machine	0.630	0.630	1.000	0.789
Multiclassification decision forest	0.891	0.831	0.730	0.777
Multiclassification neural network	0.826	0.681	0.627	0.653

The importance analysis of the 19 feature parameters in the multiclassification decision forest is shown in Supporting information S8 Table. In the RGB color model, B, G and R ranked in the top three in terms of importance for safflower quality grading, with weights of 0.241, 0.171 and 0.101, respectively. And the sum of the weights of these three feature parameters was 0.513. The color features played an important role in the process of safflower quality grading. This is in line with the description of the grade grading criteria of Chinese herbal medicines in the “Standard for Commodity Specification of 76 Medicinal Herbs", and the main difference lies in the color difference of the herbs [46]. This was followed by characteristic parameters such as KR, storage duration, a* in Lab color mode, drying temperature, and drying method. The weights of drying temperature and drying method were 0.038 and 0.035, respectively.

## Discussion

After harvesting from fresh plants, safflower composition undergoes complex changes before and after drying. The components detected by GC-MS in the fresh samples were much less than those detected in the dried samples. During the drying process, the volatile components in safflower underwent changes including oxidation and fracture, resulting in a significant increase in the total amount of volatile components after drying and the formation of the special odor of safflower herbs.

It is well known that flavonoids are the main active components of safflower. HSYA and AHSYB have a wide range of bioactive functions such as antioxidant, anti-inflammatory, anticoagulant, antitumor, and cardioprotective effects [47,48]. The FD samples contained significantly higher contents of HSYA and AHSYB than those of the other drying methods. The compositional changes during low-temperature drying at -40^∘^C were small and preserved the compositional characteristics of fresh samples of safflower. Fan et al.’s research showed that FD is more effective than NSD, SD, and OD in preserving HSYA [49]. Wang et al. and Fan et al.’s research showed that when the temperature is above 60^∘^C, HSYA and AHSYB undergo structural changes and are prone to degradation [49,50]. Yu et al. [51] and Periche et al. [52] similarly showed that FD was more effective than hot-air drying in preserving the total flavonoids. The HSYA and AHSYB contents of DD, OD, DFSD, and NSD samples were higher than those of SD samples. Flavonoid components can be decomposed by light [53]. Light exposure has a significant impact on the stability of HSYA. Under natural light conditions, the degradation rate of HSYA is significantly higher than under light-avoidance conditions. SD drying takes a longer time and is exposed to light, which is not conducive to the retention of flavonoids [54,55]. The two components, HKD and KR, were not detected in the FD, NSD, DFSD, and SD samples, however, they could be detected in the DD and OD samples. Zhou et al.’s research indicates that KR can be detected in samples processed using heating vacuum drying, oven drying, and sun drying methods, whereas it cannot be detected in vacuum freeze-dried samples [56]. The kaempferol belongs to the flavonoid alcohol class of compounds. HSYA and AHSYB can degrade under high-temperature conditions at 100^∘^C to produce flavonoid alcohol compounds [49]. Therefore, it is inferred that the thermal drying process may be conducive to the formation of HKD and KR. However, the structural transformation mechanisms of the compounds under different drying conditions require further investigation.

Macroscopic characters are important quality attributes that influence consumer acceptance of a product [57]. Temperature has a great influence on the appearance and odor properties of safflower. As the temperature increased, the types of volatile components in safflower increased, the morphology became wrinkled, and the specific odor was replaced by a burnt odor. The FD method preserves the characteristics of fresh samples better than other drying methods, resulting in a milder odor and less shrinkage. In addition, FD samples observed under SEM appear smoother overall, with clear cell arrangement. FD allowed the frozen water molecular crystals to sublimate to the gaseous state during the drying process with minimal volume shrinkage, thus better preserving the tissue structure of the samples [58–60]. The shrinkage was most pronounced in the OD samples, which could be attributed to the high moisture content of fresh safflower, and the evaporation of the water led to the collapse of the material [61]. Safflower color is an important indicator for evaluating the quality of safflower. The color of FD samples was mainly orange-yellow, followed by red, and the values of L*, a* and b* were significantly higher than those of samples from other drying methods. The physical structure of freeze-dried samples is more stereoscopic, with a more uniform distribution of colored substances. Continuous moisture loss leads to an increase in the reflectance of the sample surface, resulting in a higher L* value [62] The yellow and red pigments in safflower are relatively unstable when exposed to heat. Under low temperature and vacuum conditions, the oxidation degree of FD is low and the loss of heat-sensitive components is small, so the color of the product is better than other drying methods [63]. The ΔE* value of the OD60 sample is second only to that of the FD sample, with the lowest L*, a*, and b* values. The OD60 sample has a higher drying temperature, and compared to the OD80 sample, it requires a longer drying time to reach 13% moisture content. At this temperature, thermal degradation of the pigment molecular structure occurs, and it is insufficient to rapidly evaporate moisture, leading to continued degradation of the pigment in a prolonged hot and humid environment. Pigment degradation and physical structural collapse cause the sample color to darken and lighten, with decreases in L*, a*, and b* values. Additionally, the sample exhibits the lowest BI value, suggesting that this temperature condition primarily induces thermal degradation of safflower pigment rather than promoting enzymatic or non-enzymatic browning reactions. The higher ΔE* and lowest BI values are primarily due to pigment loss and physical darkening rather than the formation of brown substances.

The traditional empirical identification of safflower quality is mainly based on chromatic characteristics, morphological parameters, textural properties and olfactory identification based on volatile components. The modern physical and chemical identification of safflower focuses on the quantitative analysis of HSYA and other characteristic marker components. With the technical progress of modern analytical techniques, especially the wide application of HPLC, GC-MS and near infrared spectroscopy (NIRS), the quality control strategy of safflower has been gradually shifted to a multimodal detection system. Su et al. [64] used high performance liquid fingerprinting and GC-MS to objectively analyze safflower. Jia-Xi et al. [65] used ultra-high performance liquid chromatography/Q-Orbitrap mass spectrometry and nuclear magnetic resonance to determine primary and secondary metabolites to evaluate the quality of safflower. Lin et al. [66] combined computer vision and NIRS to realize rapid and nondestructive analysis of safflower. This study integrated safflower drying conditions and quality evaluation metrics to construct a safflower quality prediction model using common supervised classification methods. Compared to the two-classification logistic regression benchmark model, the multiclassification decision forest achieved the highest accuracy among all models. The integrated learning approach, when addressing the inherently complex pattern recognition challenges in multiclassification tasks, leverages variance reduction and feature importance evaluation mechanisms to maintain high generalization capability while achieving superior predictive precision. Although an expected trade-off exists between recall and F1 score, its performance on the core metric of accuracy demonstrates the architecture’s unique value in handling complex classification tasks. Moreover, it revealed intrinsic correlations between these features and quality attributes through feature importance ranking. The model identified that the RGB color features, with a cumulative weight of 0.513, were the most critical discriminative factors. This aligns closely with the traditional empirical consensus in Chinese medicinal material identification of “assessing quality through color observation [67],” validating the scientific basis and interpretability of traditional methods at a data level. Drying temperature and drying method still hold a certain weight in the model (0.038 and 0.035, respectively), indicating that processing techniques have a measurable impact on the final quality of safflower. Similarly, the two-classification decision forest also performs outstandingly in terms of accuracy and precision. The local deep support vector machine achieves a significant improvement in recall and performance balance by integrating deep feature extraction with optimal boundary determination. Compared to traditional identification methods reliant on human expertise, the established machine learning models objectively quantify multidimensional features. However, the models exhibit varying degrees of confusion in classifying different safflower grades, particularly in distinguishing between grades 2 and 4. Inherent discrepancies exist between the quantitative features relied upon by the models and the criteria used in manual identification. Substandard products may be downgraded due to discrete defects like localized mold, insect damage, or non-medicinal impurities, yet these critical details cannot be effectively captured by continuous features like overall color or active ingredient content. Conversely, these substandard samples overlap with higher-grade samples in terms of color and primary chemical composition metrics, leading the model to classify them into similar grades based solely on numerical features. This demonstrates the inherent limitations of grade prediction based solely on macroscopic physicochemical indicators when micro-defects lack quantitative characterization. To enhance model performance, future research could focus on constructing a multimodal, high-dimensional feature system. The core strategy involves integrating computer vision technology to quantify currently missing key traits, precisely calculating impurity ratios and identifying localized mold spots through image analysis. Additionally, advanced machine learning algorithms capable of capturing complex nonlinear relationships should be employed to establish robust intelligent grading models that more closely approximate the logic of classification standards.

In general, machine learning methods require large sample sizes. However, some studies have used small sample sizes. Zelic et al. [68] used applied decision tree and Bayesian classification algorithms to the diagnosis of sports injuries in 118 athletes. And Zheng et al. [69] who identified type 2 diabetes through electronic health records based on a machine learning framework in a sample of 300 patients. The sample size used in our study was limited, and further research by extending the sample size and cross-validation methods can be considered subsequently. Despite the limitations of our study, the application of machine learning methods to construct a model for safflower quality evaluation based on feature attributes is of research significance, suggesting that this approach can be generalized to other quality evaluation applications for products that require dry storage.

## Conclusion

In this study, different drying methods were used to treat safflower. Changes in chemical composition and macroscopic characters of safflower before and after drying were analyzed. The results showed that FD was beneficial in maintaining the macroscopic characters and HSYA and AHSYB components of safflower. However, the cost of FD is high and it may only be suitable for drying small batches of samples to produce high quality products. OD samples have a higher content of flavonoids, lower drying cost and controllable conditions. Considering the macroscopic characters and active ingredient contents of safflower samples, as well as the practicability, time and economic costs of actual production, oven drying below 60^∘^C is more suitable for large-scale processing and popularization in production areas. Furthermore, this study preliminarily explored the possibility of using machine learning algorithms to construct a safflower quality evaluation model and found that multiclassification decision forest performed well in predicting safflower quality. However, the universality and stability of this model still need to be further verified. Future studies can expand on this basis by increasing the sample size and range of drying conditions to further evaluate the applicability of the model.

## Supporting information

S1 Fig Drying equipment for research. (TIFF)

S2 Fig UPLC multi-component analysis of mixed standards liquid charts. (TIFF)

S1 Table Safflower samples treated with different drying methods. (DOCX)

S2 Table UPLC-Q-Orbitrap MS analysis of components in safflower. (DOCX)

S3 Table Differential compounds in different drying groups of safflower samples (n = 3). (DOCX)

S4 Table Relative content of volatile components in safflower samples. (DOCX)

S5 Table Standard curve, linearity range, and correlation coefficient for UPLC multi-component quantitative analysis. (TIFF)

S6 Table Determination of multi-component content of safflower by UPLC (n = 3). (TIFF)

S7 Table Machine learning sample information. (DOCX)

S8 Table Importance analysis of 19 feature parameters by multiclassification decision forest. (TIFF)
