# Supplementary figures and images for: Effects of drying processes on the chemical and physical properties of safflower: Towards a multidimensional quality evaluation model
Source: PLoS One. 2026 Jan 2;21(1):e0339180. doi: 10.1371/journal.pone.0339180 (PMC12758763; doi:10.1371/journal.pone.0339180)

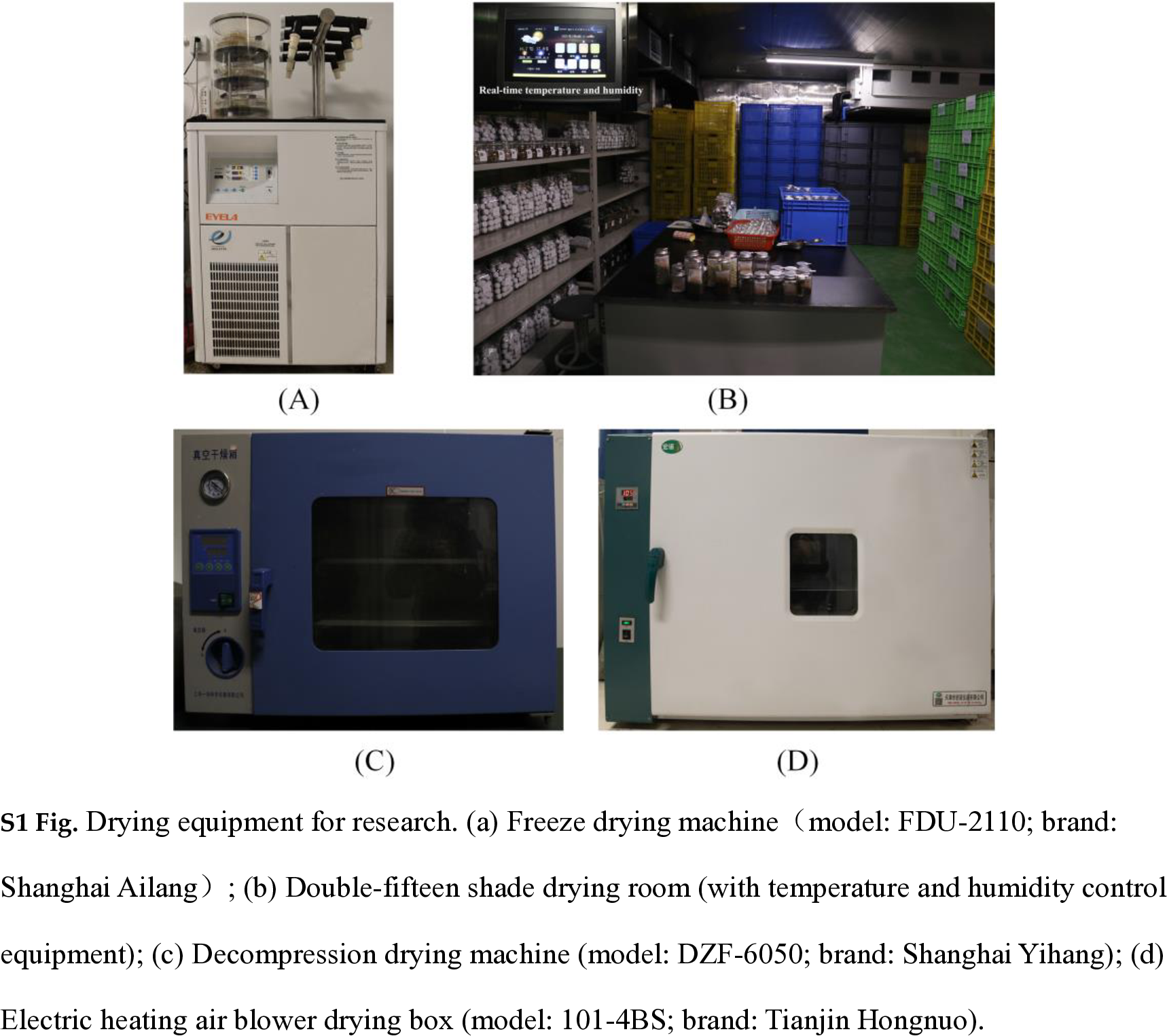

Supplement: S1 Fig — (TIFF) [file pone.0339180.s001.tif]

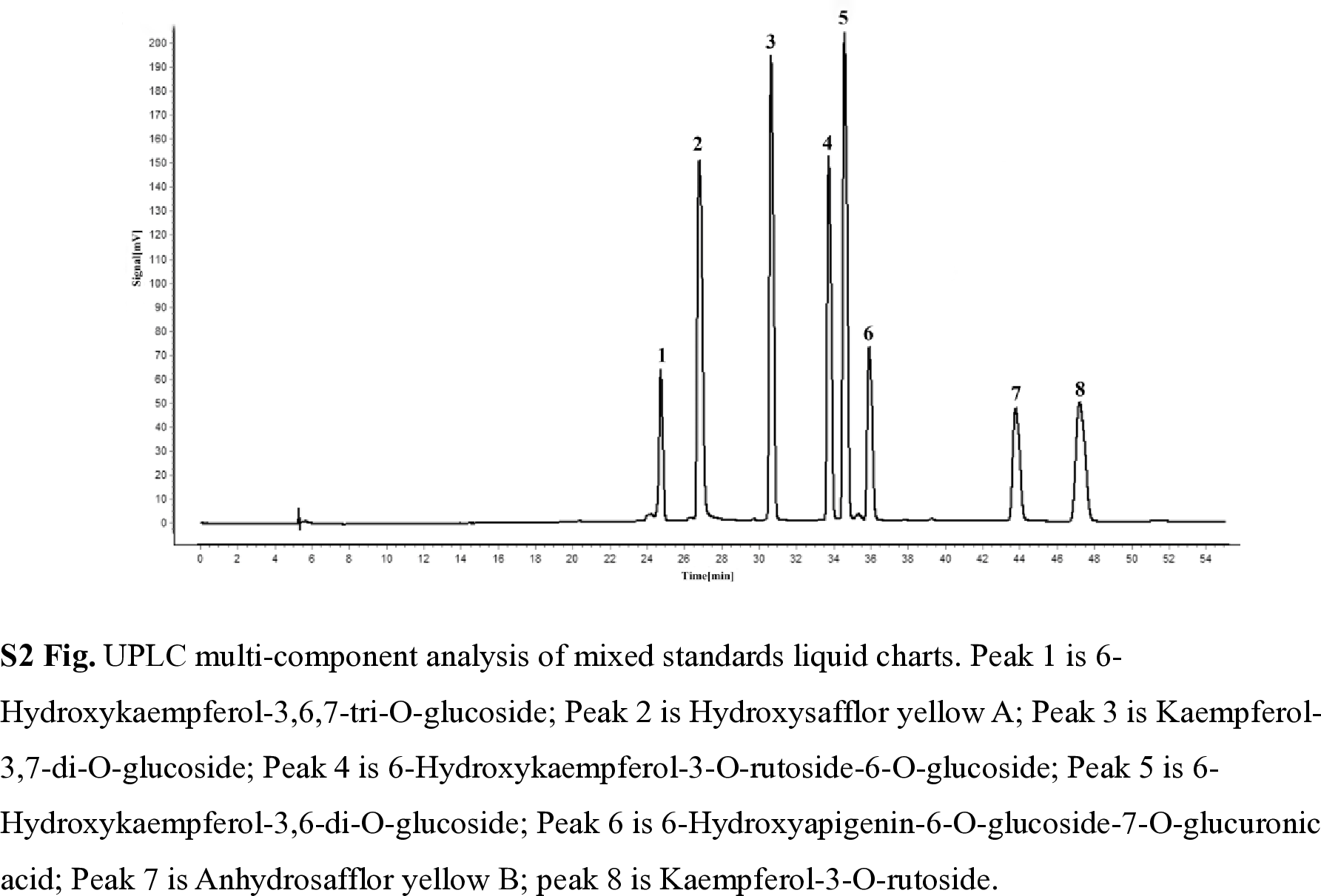

Supplement: S2 Fig — (TIFF) [file pone.0339180.s002.tif]

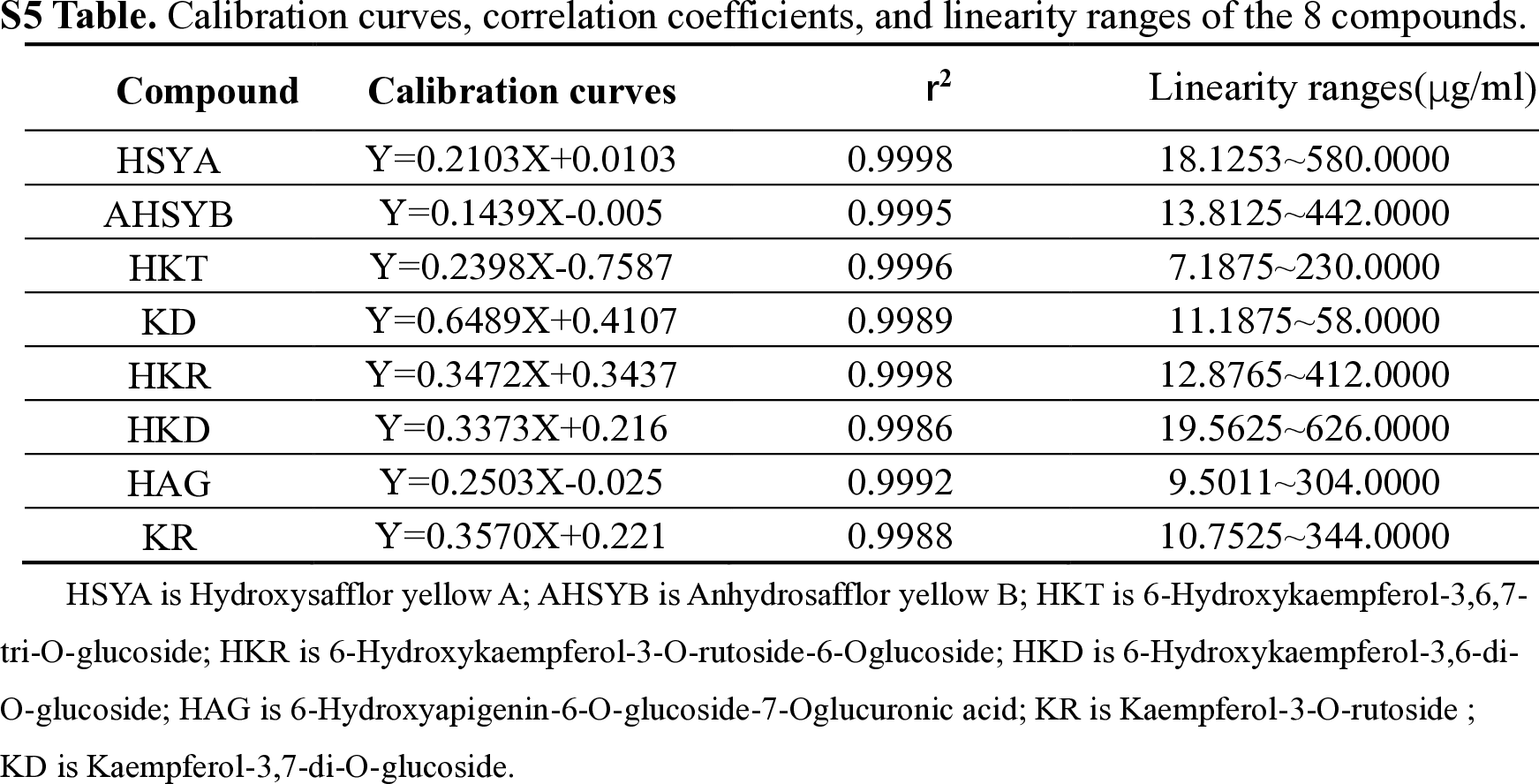

Supplement: S5 Table — (TIFF) [file pone.0339180.s007.tif]

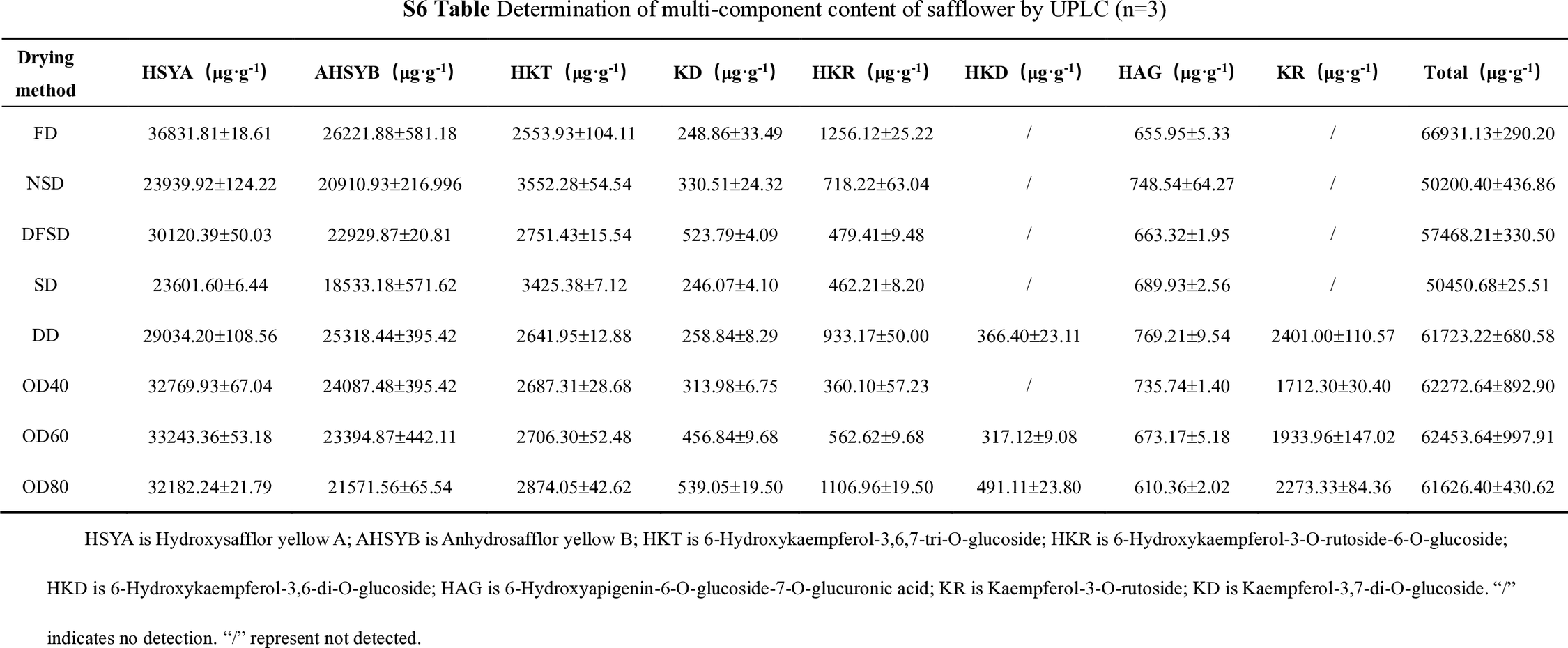

Supplement: S6 Table — (TIFF) [file pone.0339180.s008.tif]

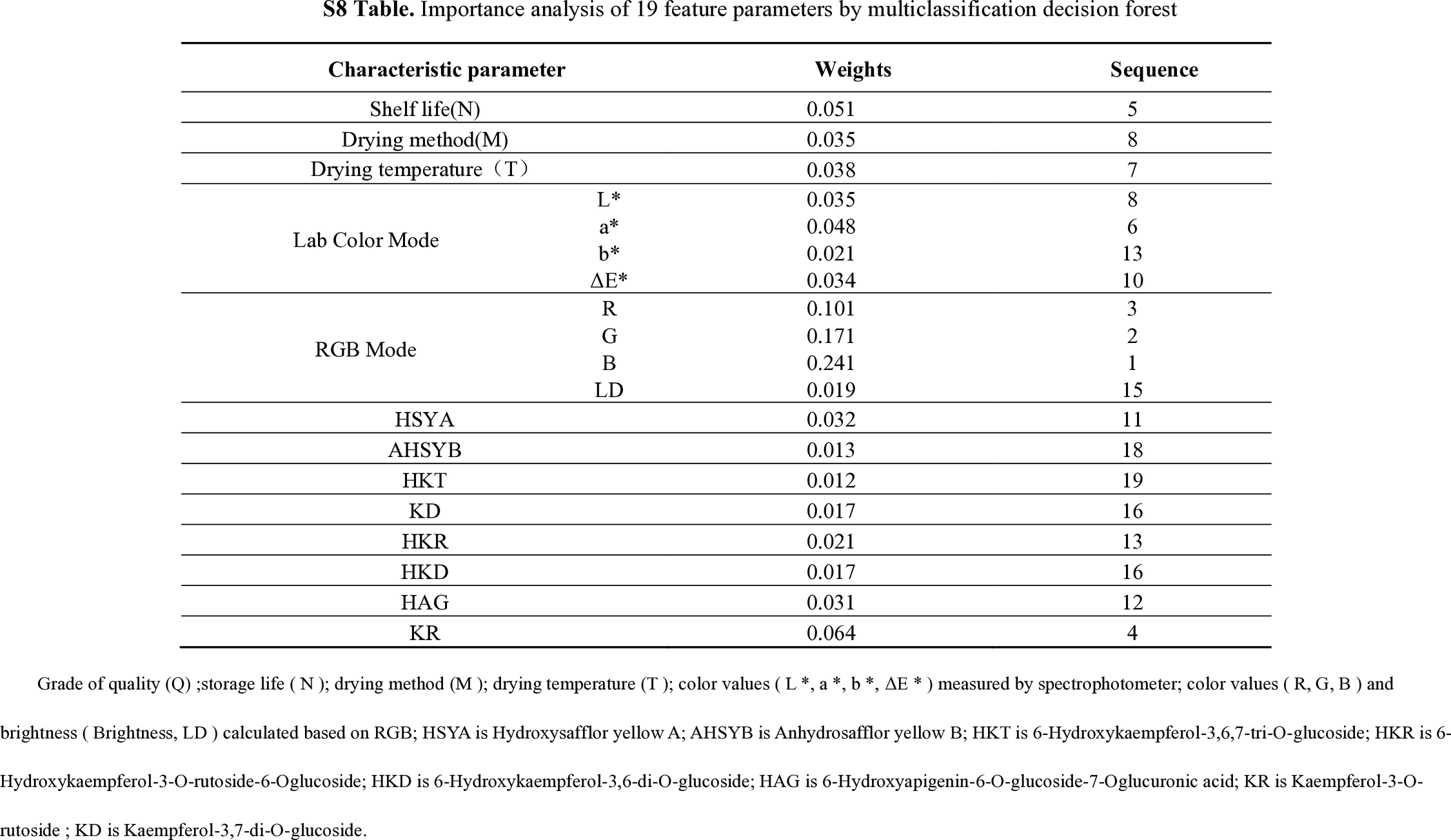

Supplement: S8 Table — (TIFF) [file pone.0339180.s010.tif]
