# Supplementary material for: Effects of drying processes on the chemical and physical properties of safflower: Towards a multidimensional quality evaluation model
Source: PLoS One. 2026 Jan 2;21(1):e0339180. doi: 10.1371/journal.pone.0339180 (PMC12758763; doi:10.1371/journal.pone.0339180)
Supplement: S1 Table — (DOCX) [file pone.0339180.s003.docx]

**S1 Table** Safflower samples treated with different drying methods.

| **Serial Number** | **drying method** | **place of origin** | **drying time** | **drying temperature（℃）** | **sunlight intensity（net,J/m2/d）** | **relative humidity（%）** | **average wind speed（m/s）** | **material acquisition methods** |
| --- | --- | --- | --- | --- | --- | --- | --- | --- |
| S1 | SD | Sichuan Province | 2~3days | 20~30℃ | 15777101 | 58.55 | 2.23 | Collection at origin |
| S2 | SD | Sichuan Province | 2~3days | 20~30℃ | 11318575 | 71.73 | 1.47 | Collection at origin |
| S3 | SD | Xinjiang Province | 2~3days | 30~45℃ | 20486025 | 57.29 | 2.00 | Collection at origin |
| S4 | SD | Xinjiang Province | 2~3days | 30~45℃ | 19819591 | 28.37 | 4.14 | Collection at origin |
| S5 | SD | Xinjiang Province | 2~3days | 30~45℃ | 20628382 | 40.73 | 2.90 | Collection at origin |
| S6 | SD | Xinjiang Province | 2~3days | 30~45℃ | 18798733 | 28.47 | 3.73 | Collection at origin |
| S7 | SD | Xinjiang Province | 2~3days | 30~45℃ | 18798733 | 28.47 | 3.73 | Collection at origin |
| S8 | SD | Sichuan Province | 2~3days | 20~30℃ | 11879766 | 76.94 | 1.50 | Collection at origin |
| S9 | SD | Gansu Province | 2~3days | 30~45℃ | 19168694 | 29.76 | 4.18 | Collection at origin |
| S10 | SD | Gansu Province | 2~3days | 30~45℃ | 19046494 | 30.87 | 4.34 | Collection at origin |
| S11 | SD | Xinjiang Province | 2~3days | 30~45℃ | - | - | - | Purchased at the market |
| S12 | SD | Xinjiang Province | 2~3days | 30~45℃ | - | - | - | Purchased at the market |
| S13 | SD | Xinjiang Province | 2~3days | 30~45℃ | - | - | - | Purchased at the market |
| S14 | SD | Xinjiang Province | 2~3days | 30~45℃ | - | - | - | Purchased at the market |
| S15 | SD | Xinjiang Province | 2~3days | 30~45℃ | - | - | - | Purchased at the market |
| S16 | SD | Xinjiang Province | 2~3days | 30~45℃ | - | - | - | Purchased at the market |
| S17 | SD | Xinjiang Province | 2~3days | 30~45℃ | - | - | - | Purchased at the market |
| S18 | SD | Xinjiang Province | 2~3days | 30~45℃ | - | - | - | Purchased at the market |
| S19 | SD | Xinjiang Province | 2~3days | 30~45℃ | - | - | - | Purchased at the market |
| S20 | SD | Xinjiang Province | 2~3days | 30~45℃ | - | - | - | Purchased at the market |
| S21 | SD | Xinjiang Province | 2~3days | 30~45℃ | 19432147 | 40.52 | 2.86 | Purchased at the market |
| S22 | SD | Yunnan Province | 2~3days | 25~35℃ | 20386965 | 32.10 | 2.85 | Purchased at the market |
| S23 | SD | Sichuan Province | 2~3days | 20~30℃ | 15457938 | 47.05 | 2.45 | Collection at origin |
| S24 | SD | Sichuan Province | 2~3days | 20~30℃ | - | - | - | Purchased at the market |
| S25 | SD | Sichuan Province | 2~3days | 20~30℃ | - | - | - | Purchased at the market |
| S26 | SD | Sichuan Province | 2~3days | 20~30℃ | 12634018 | 70.94 | 1.89 | Collection at origin |
| S27 | SD | Yunnan Province | 2~3days | 25~35℃ | 19339426 | 32.94 | 2.94 | Collection at origin |
| S28 | SD | Yunnan Province | 2~3days | 25~35℃ | 19335995 | 30.88 | 4.23 | Collection at origin |
| S29 | SD | Yunnan Province | 2~3days | 25~35℃ | 18798855 | 31.69 | 3.62 | Collection at origin |
| S30 | OD | Henan Province | 13h | 30~50℃ | 17455582 | 62.09 | 3.27 | Collection at origin |
| S31 | OD | Henan Province | 13h | 30~50℃ | 17633158 | 58.62 | 3.32 | Collection at origin |
| S32 | OD | Henan Province | 13h | 30~50℃ | 17537949 | 60.76 | 3.30 | Collection at origin |
| S33 | OD | Henan Province | 13h | 30~50℃ | 18457712 | 54.44 | 3.49 | Collection at origin |
| S34 | SD | Gansu Province | 2~3days | 30~45℃ | 18951210 | 30.49 | 4.86 | Collection at origin |
| S35 | SD | Gansu Province | 2~3days | 30~45℃ | 19353931 | 47.21 | 2.75 | Collection at origin |
| S36 | SD | Gansu Province | 2~3days | 30~45℃ | 19402308 | 28.49 | 3.97 | Collection at origin |
| S37 | SD | Xinjiang Province | 2~3days | 30~45℃ | 19460022 | 36.91 | 2.85 | Collection at origin |
| S38 | SD | Xinjiang Province | 2~3days | 30~45℃ | 19460022 | 36.91 | 2.85 | Collection at origin |
| S39 | SD | Xinjiang Province | 2~3days | 30~45℃ | 19570780 | 44.00 | 2.03 | Collection at origin |
| S40 | SD | Xinjiang Province | 2~3days | 30~45℃ | 19622553 | 41.67 | 2.30 | Collection at origin |
| S41 | NSD | Sichuan Province | 72h | 20℃ | - | 72.21 | - | Prepared in the laboratory |
| S42 | DFSD | Sichuan Province | 50h | 15℃ | - | 15 | - | Prepared in the laboratory |
| S43 | SD | Sichuan Province | 48h | 28℃ | 12327578 | 72.21 | 1.53 | Prepared in the laboratory |
| S44 | FD | Sichuan Province | 9h | -40℃ | - | - | - | Prepared in the laboratory |
| S45 | DD | Sichuan Province | 30h | 30℃ | - | - | - | Prepared in the laboratory |
| S46 | OD40 | Sichuan Province | 6h | 40℃ | - | - | - | Prepared in the laboratory |
| S47 | OD60 | Sichuan Province | 3h | 60℃ | - | - | - | Prepared in the laboratory |
| S48 | OD80 | Sichuan Province | 1.5h | 80℃ | - | - | - | Prepared in the laboratory |

“-” represents information that could not be captured. This table lists the basic information for 48 independent biological samples. Each sample has 3 biological replicates, with all 144 data points used for subsequent modeling analysis.
