# Supplementary material for: Effects of drying processes on the chemical and physical properties of safflower: Towards a multidimensional quality evaluation model
Source: PLoS One. 2026 Jan 2;21(1):e0339180. doi: 10.1371/journal.pone.0339180 (PMC12758763; doi:10.1371/journal.pone.0339180)
Supplement: S2 Table — (DOCX) [file pone.0339180.s004.docx]

**S2 Table** UPLC-Q-Orbitrap MS analysis of components in safflower

| NO. | Name | Formula | Molecular Weight | RT [min] | classification | Ion Type |
| --- | --- | --- | --- | --- | --- | --- |
| 1 | L-Lysine | C6H14N2O2 | 146.11 | 2.12 | Amino acids and derivatives | [M-H]^-^ |
| 2 | L-Histidine | C6H9N3O2 | 155.07 | 2.14 | Amino acids and derivatives | [M+H]^+^ |
| 3 | N6,N6,N6-Trimethyl-L-lysine | C9H20N2O2 | 188.15 | 2.16 | Amino acids and derivatives | [M+H]^+^ |
| 4 | DL-Arginine | C6H14N4O2 | 174.11 | 2.17 | Amino acids and derivatives | [M+H]^+^ |
| 5 | D-Glucosamine | C6H13NO5 | 179.08 | 2.22 | carbohydrates and derivatives | [M-H]^-^ |
| 6 | Kanosamine | C6H13NO5 | 161.07 | 2.22 | carbohydrates and derivatives | [M+H]^+^ |
| 7 | L-Serine | C3H7NO3 | 105.04 | 2.25 | Amino acids and derivatives | [M+H]^+^ |
| 8 | Asparagine | C4H8N2O3 | 132.05 | 2.26 | Amino acids and derivatives | [M+H]^+^ |
| 9 | D-(-)-Glutamine | C5H10N2O3 | 146.07 | 2.27 | Amino acids and derivatives | [M+H]^+^ |
| 10 | Choline | C5H13NO | 103.10 | 2.27 | lipids | [M+H]^+^ |
| 11 | D-FRUCTOSE | C6H12O6 | 180.06 | 2.28 | carbohydrates and derivatives | [M-H]^-^ |
| 12 | D-Serine | C3H7NO3 | 105.04 | 2.28 | Amino acids and derivatives | [M+H]^+^ |
| 13 | L-Pyroglutamic acid | C5H7NO3 | 129.04 | 2.30 | Amino acids and derivatives | [M-H]^-^ |
| 14 | L-Aspartic acid | C4H7NO4 | 133.04 | 2.30 | Amino acids and derivatives | [M+H]^+^ |
| 15 | Threonine | C4H9NO3 | 119.06 | 2.31 | Amino acids and derivatives | [M+H]^+^ |
| 16 | Phosphocholine | C5H14NO4P | 183.07 | 2.31 | lipids | [M+H]^+^ |
| 17 | Cytosine | C4H5N3O | 111.04 | 2.32 | Nucleosides, nucleotides and derivatives | [M+H]^+^ |
| 18 | Muramic acid | C9H17NO7 | 251.10 | 2.32 | carbohydrates and derivatives | [M-H]^-^ |
| 19 | 4-aminobutanoate | C4H9NO2 | 103.06 | 2.32 | Amino acids and derivatives | [M+H]^+^ |
| 20 | cis-4-Hydroxy-D-proline | C5H9NO3 | 131.06 | 2.32 | Amino acids and derivatives | [M-H]^-^ |
| 21 | L-Glutamic acid | C5H9NO4 | 147.05 | 2.35 | Amino acids and derivatives | [M+H]^+^ |
| 22 | D-Saccharic acid | C6H10O8 | 210.04 | 2.35 | carbohydrates and derivatives | [M-H]^-^ |
| 23 | DL-Carnitine | C7H15NO3 | 161.11 | 2.35 | Amino acids and derivatives | [M-H]^-^ |
| 24 | Methionine sulfoxide | C5H11NO3S | 148.02 | 2.35 | Amino acids and derivatives | [M+H]^+^ |
| 25 | Galacturonic acid | C6H10O7 | 194.04 | 2.36 | carbohydrates and derivatives | [M-H]^-^ |
| 26 | Gluconic acid | C6H12O7 | 196.06 | 2.36 | carbohydrates and derivatives | [M-H]^-^ |
| 27 | Triethanolamine | C6H15NO3 | 149.11 | 2.37 | amines | [M-H]^-^ |
| 28 | Betaine | C5H11NO2 | 117.08 | 2.39 | lipids | [M+H]^+^ |
| 29 | D-(-)-Fructose | C6H12O6 | 180.06 | 2.42 | carbohydrates and derivatives | [M-H]^-^ |
| 30 | L-Threonic acid | C4H8O5 | 136.04 | 2.44 | organic acid | [M-H]^-^ |
| 31 | D-(+)-Proline | C5H9NO2 | 115.06 | 2.45 | Amino acids and derivatives | [M+H]^+^ |
| 32 | Trigonelline | C7H7NO2 | 137.05 | 2.45 | alkaloids | [M-H]^-^ |
| 33 | L-2-Aminoadipic acid | C6H11NO4 | 161.07 | 2.45 | Amino acids and derivatives | [M-H]^-^ |
| 34 | D-(-)-Quinic acid | C7H12O6 | 192.06 | 2.47 | organic acid | [M+H]^+^ |
| 35 | δ-Gluconic acid δ-lactone | C6H10O6 | 178.05 | 2.53 | carbohydrates and derivatives | [M-H]^-^ |
| 36 | DL-Stachydrine | C7H13NO2 | 143.10 | 2.54 | alkaloids | [M-H]^-^ |
| 37 | Shikimic Acid | C7H10O5 | 174.05 | 2.74 | organic acid | [M+H]^+^ |
| 38 | Adenine | C5H5N5 | 135.05 | 2.75 | Nucleosides, nucleotides and derivatives | [M-H]^-^ |
| 39 | L-Valine | C5H11NO2 | 117.08 | 2.75 | Amino acids and derivatives | [M-H]^-^ |
| 40 | 4-Guanidinobutyric acid | C5H11N3O2 | 145.09 | 2.80 | Amino acids and derivatives | [M+H]^+^ |
| 41 | Isocitric acid | C6H8O7 | 192.03 | 2.83 | organic acid | [M-H]^-^ |
| 42 | DL-Malic acid | C4H6O5 | 134.02 | 2.85 | organic acid | [M-H]^-^ |
| 43 | Urocanic acid | C6H6N2O2 | 138.04 | 2.89 | organic acid | [M+H]^+^ |
| 44 | 1-Methyladenosine | C11H15N5O4 | 281.11 | 2.91 | Nucleosides, nucleotides and derivatives | [M+H]^+^ |
| 45 | Malonic acid | C3H4O4 | 104.01 | 3.02 | organic acid | [M+H]^+^ |
| 46 | Acetyl-L-carnitine | C9H17NO4 | 203.12 | 3.04 | Amino acids and derivatives | [M-H]^-^ |
| 47 | 3-DEHYDROSHIKIMATE | C7H8O5 | 172.04 | 3.08 | organic acid | [M+H]^+^ |
| 48 | Uric acid | C5H4N4O3 | 168.03 | 3.12 | organic acid | [M-H]^-^ |
| 49 | L-Glutathione (reduced) | C10H17N3O6S | 307.08 | 3.17 | peptide | [M+H]^+^ |
| 50 | Nicotinic acid | C6H5NO2 | 123.03 | 3.24 | vitamin | [M-H]^-^ |
| 51 | Methionine | C5H11NO2S | 149.05 | 3.29 | Amino acids and derivatives | [M-H]^-^ |
| 52 | 5-Methylcytosine | C5H7N3O | 125.06 | 3.32 | Nucleosides, nucleotides and derivatives | [M-H]^-^ |
| 53 | 7-Methylguanosine | C11H15N5O5 | 297.11 | 3.32 | Nucleosides, nucleotides and derivatives | [M+H]^+^ |
| 54 | L-Glutathione oxidized | C20H32N6O12S2 | 612.15 | 3.33 | peptide | [M+H]^+^ |
| 55 | Hypoxanthine | C5H4N4O | 136.04 | 3.35 | alkaloids | [M+H]^+^ |
| 56 | D-α-Hydroxyglutaric acid | C5H8O5 | 148.04 | 3.35 | organic acid | [M-H]^-^ |
| 57 | Citric acid | C6H8O7 | 192.03 | 3.40 | organic acid | [M-H]^-^ |
| 58 | Glutaconic acid | C5H6O4 | 130.03 | 3.41 | organic acid | [M-H]^-^ |
| 59 | 6-Hydroxynicotinic acid | C6H5NO3 | 139.03 | 3.41 | organic acid | [M-H]^-^ |
| 60 | Uridine | C9H12N2O6 | 244.07 | 3.52 | Nucleosides, nucleotides and derivatives | [M-H]^-^ |
| 61 | Uracil | C4H4N2O2 | 112.03 | 3.55 | Nucleosides, nucleotides and derivatives | [M-H]^-^ |
| 62 | 4-Oxoproline | C5H7NO3 | 129.04 | 3.56 | Amino acids and derivatives | [M-H]^-^ |
| 63 | Xanthine | C5H4N4O2 | 152.03 | 3.58 | alkaloids | [M+H]^+^ |
| 64 | 2-Hydroxyphenylalanine | C9H11NO3 | 181.07 | 3.79 | Amino acids and derivatives | [M+H]^+^ |
| 65 | 2-Hydroxycinnamic acid | C9H8O3 | 164.05 | 4.00 | organic acid | [M+H]^+^ |
| 66 | Acetophenone | C8H8O | 120.06 | 4.09 | - | [M+H]^+^ |
| 67 | Fumaric acid | C4H4O4 | 116.01 | 4.14 | organic acid | [M-H]^-^ |
| 68 | Citramalic acid | C5H8O5 | 148.04 | 4.21 | organic acid | [M-H]^-^ |
| 69 | 2'-Deoxyadenosine | C10H13N5O3 | 251.10 | 4.28 | Nucleosides, nucleotides and derivatives | [M+H]^+^ |
| 70 | Tyramine | C8H11NO | 137.08 | 4.28 | amines | [M-H]^-^ |
| 71 | DL-Norleucine | C6H13NO2 | 131.10 | 4.29 | Amino acids and derivatives | [M-H]^-^ |
| 72 | Succinic acid | C4H6O4 | 118.03 | 4.36 | organic acid | [M+H]^+^ |
| 73 | Guanine | C5H5N5O | 151.05 | 4.49 | Nucleosides, nucleotides and derivatives | [M+H]^+^ |
| 74 | Guanosine | C10H13N5O5 | 283.09 | 4.49 | Nucleosides, nucleotides and derivatives | [M-H]^-^ |
| 75 | Thymine | C5H6N2O2 | 126.04 | 4.85 | Nucleosides, nucleotides and derivatives | [M-H]^-^ |
| 76 | Gly-Ile | C8H16N2O3 | 188.12 | 4.90 | peptide | [M+H]^+^ |
| 77 | 2'-O-Methyladenosine | C11H15N5O4 | 281.11 | 6.66 | Nucleosides, nucleotides and derivatives | [M+H]^+^ |
| 78 | L-Phenylalanine | C9H11NO2 | 148.05 | 7.41 | Amino acids and derivatives | [M+H]^+^ |
| 79 | Pantothenic acid | C9H17NO5 | 219.11 | 8.66 | vitamin | [M+H]^+^ |
| 80 | Glycyl-L-leucine | C8H16N2O3 | 188.12 | 8.86 | peptide | [M-H]^-^ |
| 81 | PEG n5 | C10H22O6 | 238.14 | 10.46 | - | [M-H]^-^ |
| 82 | 2,3-Dihydroxybenzoic acid | C7H6O4 | 154.03 | 10.46 | organic acid | [M-H]^-^ |
| 83 | Leucylproline | C11H20N2O3 | 228.15 | 10.73 | peptide | [M-H]^-^ |
| 84 | Indole-3-acrylic acid | C11H9NO2 | 187.06 | 11.16 | organic acid | [M+H]^+^ |
| 85 | 4-Indolecarbaldehyde | C9H7NO | 145.05 | 11.16 | aldehydes | [M+H]^+^ |
| 86 | DL-Tryptophan | C11H12N2O2 | 204.09 | 11.16 | Amino acids and derivatives | [M+H]^+^ |
| 87 | Melilotoside | C_15_H_18_O_8_ | 372.11 | 11.22 | flavonoids | [M+H]^+^ |
| 88 | 3-[3-[4,5-dihydroxy-6-(hydroxymethyl)-3-[3,4,5-trihydroxy-6-(hydroxymethyl)oxan-2-yl]oxyoxan-2-yl]oxy-4,5-dihydroxy-6-(hydroxymethyl)oxan-2-yl]oxy-2-(3,4-dihydroxyphenyl)-5,7-dihydroxychromen-4-one | C33H40O22 | 788.20 | 11.64 | flavonoids | [M-H]^-^ |
| 89 | PEG n6 | C12H26O7 | 282.17 | 11.99 | - | [M+H]^+^ |
| 90 | Hydroxysafflor Yellow A | C27H32O16 | 612.17 | 12.57 | flavonoids | [M+H]^+^ |
| 91 | 2-Isopropylmalic acid | C7H12O5 | 176.07 | 12.79 | organic acid | [M-H]^-^ |
| 92 | Eleutheroside B/Syringin | C17H24O9 | 389.17 | 12.80 | flavonoids | [M+H]^+^ |
| 93 | Chlorogenic acid | C16H18O9 | 354.10 | 13.21 | organic acid | [M+H]^+^ |
| 94 | PEG n7 | C14H30O8 | 326.19 | 13.21 | - | [M+H]^+^ |
| 95 | N-Valylphenylalanine | C14H20N2O3 | 264.15 | 13.76 | peptide | [M+H]^+^ |
| 96 | Salicylic acid | C7H6O3 | 138.03 | 13.82 | organic acid | [M-H]^-^ |
| 97 | 2,3,4,9-Tetrahydro-1H-β-carboline-3-carboxylic acid | C12H12N2O2 | 216.09 | 14.14 | alkaloids | [M-H]⁻ |
| 98 | PEG n8 | C16H34O9 | 370.22 | 14.32 | - | [M-H]^-^ |
| 99 | 3-[3-(beta-D-Glucopyranosyloxy)-2-methoxyphenyl]propanoic acid | C16H22O9 | 358.13 | 15.06 | carbohydrates and derivatives | [M-H]⁻ |
| 100 | 3-Hydroxy-3,5,5-trimethyl-4-(3-oxo-1-buten-1-ylidene)cyclohexyl β-D-glucopyranoside | C19H30O8 | 432.20 | 15.21 | carbohydrates and derivatives | [M-H]⁻ |
| 101 | Caffeic acid | C9H8O4 | 180.04 | 15.27 | organic acid | [M+H]^+^ |
| 102 | NCGC00385380-01! | C27H30O17 | 626.15 | 16.11 | flavonoids | [M+H]^+^ |
| 103 | PEG n10 | C20H42O11 | 475.30 | 16.94 | - | [M-H]^-^ |
| 104 | PEG n11 | C22H46O12 | 519.33 | 17.85 | - | [M-H]^-^ |
| 105 | {3-[(3-{[4-(Hydroxymethyl)cyclohexyl]amino}-3-oxetanyl)methyl]-1,2-oxazol-5-yl}methanol | C15H24N2O4 | 278.16 | 18.16 | - | [M+H]⁺ |
| 106 | 5,6,7,8-Tetrahydro-2-naphthol | C10H12O | 148.09 | 18.91 | - | [M-H]⁻ |
| 107 | PEG n12 | C24H50O13 | 563.35 | 18.98 | - | [M-H]^-^ |
| 108 | 3-Coumaric acid | C9H8O3 | 164.05 | 19.53 | organic acid | [M-H]^-^ |
| 109 | Rutin | C27H30O16 | 610.15 | 19.94 | flavonoids | [M-H]^-^ |
| 110 | Quercetin 3-O-rhamnoside-7-O-glucoside | C27H30O16 | 610.15 | 20.10 | flavonoids | [M-H]^-^ |
| 111 | PEG n13 | C26H54O14 | 607.38 | 20.14 | - | [M-H]^-^ |
| 112 | Quercetin-3β-D-glucoside | C21H20O12 | 464.10 | 21.51 | flavonoids | [M-H]^-^ |
| 113 | Kaempferol | C15H10O6 | 286.05 | 22.69 | flavonoids | [M+H]^+^ |
| 114 | Trifolin | C21H20O11 | 448.10 | 22.69 | flavonoids | [M-H]^-^ |
| 115 | Scutellarin | C21H18O12 | 462.08 | 25.21 | flavonoids | [M+H]^+^ |
| 116 | Apigenin 7-O-glucuronide | C21H18O11 | 446.09 | 25.44 | flavonoids | [M+H]^+^ |
| 117 | Azelaic acid | C9H16O4 | 188.10 | 26.52 | organic acid | [M+H]^+^ |
| 118 | Quercetin | C15H10O7 | 302.04 | 30.75 | flavonoids | [M+H]^+^ |
| 119 | Ligustilide | C12H14O2 | 190.10 | 30.95 | terpenoid | [M+H]^+^ |
| 120 | Nootkatone | C15H22O | 218.17 | 31.13 | terpenoid | [M+H]^+^ |
| 121 | 9-Oxo-10E,12Z-octadecadienoic acid | C18H30O3 | 294.22 | 31.24 | lipids | [M+H]^+^ |
| 122 | 9S,13R-12-Oxophytodienoic acid | C18H28O3 | 292.20 | 31.24 | organic acid | [M+H]^+^ |
| 123 | (11E,15Z)-9,10,13-trihydroxyoctadeca-11,15-dienoic acid | C18H32O5 | 345.25 | 31.28 | lipids | [M-H]⁻ |
| 124 | Corchorifatty acid F | C18H32O5 | 328.23 | 31.30 | lipids | [M+H]^+^ |
| 125 | 12,13-dihydroxyoctadec-9-enoic acid | C18H34O4 | 296.24 | 31.68 | lipids | [M-H]⁻ |
| 126 | N,N-Dimethyldecylamine N-oxide | C12H27NO | 201.21 | 31.73 | amines | [M-H]^-^ |
| 127 | Genistein | C15H10O5 | 270.05 | 31.81 | flavonoids | [M+H]^+^ |
| 128 | (9Z,11E,13S,15Z)-13-hydroxyoctadeca-9,11,15-trienoic acid | C18H30O3 | 294.22 | 31.82 | lipids | [M+H]^+^ |
| 129 | (15Z)-9,12,13-Trihydroxy-15-octadecenoic acid | C18H34O5 | 330.24 | 31.83 | lipids | [M+H]^+^ |
| 130 | (4S,4aR)-4-(hydroxymethyl)-3,4a,8,8-tetramethyl-5,6,7,8a-tetrahydro-4H-naphthalen-1-one | C15H24O2 | 236.18 | 32.18 | terpenoid | [M+H]^+^ |
| 131 | Pentadecanoic Acid | C15H30O2 | 288.23 | 32.38 | lipids | [M-H]^-^ |
| 132 | 4-methylidene-2-octyl-5-oxooxolane-3-carboxylic acid | C14H22O4 | 254.15 | 33.93 | organic acid | [M-H]⁻ |
| 133 | Perfluorooctanoic acid (PFOA) | C8HF15O2 | 413.97 | 34.05 | organic acid | [M-H]^-^ |
| 134 | Methyl palmitate | C17H34O2 | 287.28 | 34.47 | lipids | [M+H]^+^ |
| 135 | (10E,12Z)-9-hydroperoxyoctadeca-10,12-dienoic acid | C18H32O4 | 312.23 | 35.32 | lipids | [M-H]⁻ |
| 136 | Didecyldimethylammonium | C22H47N | 325.37 | 35.63 | amines | [M-H]^-^ |
| 137 | Dodecyl sulfate | C12H26O4S | 266.16 | 35.89 | lipids | [M+H]^+^ |
| 138 | Benzophenone | C13H10O | 182.07 | 35.94 | - | [M+H]^+^ |
| 139 | Bis(4-ethylbenzylidene)sorbitol | C24H30O6 | 414.20 | 35.99 | - | [M+H]^+^ |
| 140 | 2-Amino-1,3,4-octadecanetriol | C18H39NO3 | 317.29 | 36.10 | - | [M-H]^-^ |
| 141 | Oleamide | C18H35NO | 281.27 | 36.26 | lipids | [M+H]^+^ |
| 142 | Cuminaldehyde | C10H12O | 148.09 | 36.62 | aldehydes | [M-H]^-^ |
| 143 | Oxybenzone | C14H12O3 | 228.08 | 36.75 | - | [M+H]^+^ |
| 144 | 4-Methoxycinnamic acid | C10H10O3 | 178.06 | 37.65 | organic acid | [M+H]^+^ |
| 145 | Stearamide | C18H37NO | 283.29 | 37.69 | lipids | [M+H]^+^ |
| 146 | Myristyl sulfate | C14H30O4S | 294.19 | 38.17 | lipids | [M-H]^-^ |
| 147 | Diisobutylphthalate | C16H22O4 | 278.15 | 38.84 | lipids | [M+H]^+^ |
| 148 | Dibutyl phthalate | C16H22O4 | 278.15 | 38.95 | lipids | [M+H]^+^ |
| 149 | Dibutyl sebacate | C18H34O4 | 314.25 | 40.01 | lipids | [M+H]^+^ |
| 150 | Linoleoyl ethanolamide | C20H37NO2 | 323.28 | 40.79 | lipids | [M-H]^-^ |
| 151 | Erucamide | C22H43NO | 337.34 | 40.83 | lipids | [M+H]^+^ |
| 152 | Bis(2-ethylhexyl) amine | C16H35N | 241.28 | 40.92 | amines | [M+H]^+^ |
| 153 | Hexadecanamide | C16H33NO | 255.26 | 41.88 | lipids | [M+H]^+^ |
| 154 | Octadecanamine | C18H39N | 269.31 | 42.19 | lipids | [M+H]^+^ |
| 155 | 3-[(Carboxycarbonyl)amino]-L-alanine | C5H8N2O5 | 175.96 | 43.07 | Amino acids and derivatives | [M-H]⁻ |
