# Supplementary material for: Effects of drying processes on the chemical and physical properties of safflower: Towards a multidimensional quality evaluation model
Source: PLoS One. 2026 Jan 2;21(1):e0339180. doi: 10.1371/journal.pone.0339180 (PMC12758763; doi:10.1371/journal.pone.0339180)
Supplement: S3 Table — (DOCX) [file pone.0339180.s005.docx]

**S3 Table** Differential compounds in different drying groups of safflower samples (n=3)

| NO. | Name | Formula | RT [min] | VIP | log_2_Foldchange | | | | | | | |
| --- | --- | --- | --- | --- | --- | --- | --- | --- | --- | --- | --- | --- |
|  |  |  |  |  | **OD40/F** | **OD60/F** | **OD80/F** | **FD/F** | **DD/F** | **SD/F** | **DFSD/F** | **NSD/F** |
| 1 | Phosphocholine | C5H14NO4P | 2.31 | 1.17 | -4.77 | -4.35 | -3.28 | -8.44 | -4.66 | -9.23 | -6.86 | -8.55 |
| 2 | Muramic acid | C9H17NO7 | 2.32 | 1.16 | 4.29 | 5.39 | 6.18 | 4.64 | 4.70 | 3.05 | 5.15 | 4.86 |
| 3 | L-Glutamic acid | C5H9NO4 | 2.35 | 1.13 | 1.25 | 1.84 | 2.53 | 1.36 | 1.57 | -0.04 | 2.31 | 1.84 |
| 4 | D-Saccharic acid | C6H10O8 | 2.35 | 1.33 | -3.50 | -2.78 | -2.81 | -3.50 | -1.72 | -5.56 | -1.45 | -4.28 |
| 5 | Galacturonic acid | C6H10O7 | 2.36 | 1.23 | 0.42 | 0.31 | -0.11 | 1.65 | 0.55 | 0.64 | 0.90 | 1.38 |
| 6 | Triethanolamine | C6H15NO3 | 2.37 | 1.27 | -7.79 | -8.09 | -7.91 | -4.62 | -8.46 | -8.31 | -8.82 | -8.87 |
| 7 | D-(-)-Quinic acid | C7H12O6 | 2.47 | 1.03 | -1.20 | -0.66 | -0.70 | -0.39 | -1.03 | -2.65 | -1.27 | -1.74 |
| 8 | δ-Gluconic acid δ-lactone | C6H10O6 | 2.53 | 1.13 | 1.17 | 1.38 | 1.30 | 1.76 | 1.58 | 1.43 | 1.83 | 2.76 |
| 9 | 4-Guanidinobutyric acid | C5H11N3O2 | 2.80 | 1.03 | 0.09 | 0.36 | 0.33 | 0.39 | 0.31 | 0.25 | 0.97 | 1.24 |
| 10 | Malonic acid | C3H4O4 | 3.02 | 1.07 | 1.42 | 1.73 | 1.42 | 1.51 | 1.18 | -5.59 | 1.88 | 2.34 |
| 11 | Acetyl-L-carnitine | C9H17NO4 | 3.04 | 1.11 | 0.51 | 0.79 | 0.49 | 2.20 | 0.87 | 1.50 | -0.34 | 0.30 |
| 12 | 3-DEHYDROSHIKIMATE | C7H8O5 | 3.08 | 1.14 | -3.89 | -3.88 | -5.13 | -2.79 | -6.52 | -6.58 | -7.18 | -7.58 |
| 13 | L-Glutathione (reduced) | C10H17N3O6S | 3.17 | 1.08 | -1.65 | -1.01 | -1.29 | -1.13 | -1.77 | -3.12 | -1.41 | -1.05 |
| 14 | L-Glutathione oxidized | C20H32N6O12S2 | 3.33 | 1.08 | -2.68 | -2.59 | -2.96 | -2.52 | -1.64 | -9.59 | -1.46 | -2.27 |
| 15 | Hypoxanthine | C5H4N4O | 3.35 | 1.19 | -0.08 | 0.32 | -1.01 | 0.34 | 0.37 | 0.70 | -1.23 | -0.31 |
| 16 | D-α-Hydroxyglutaric acid | C5H8O5 | 3.35 | 1.02 | -1.41 | -1.14 | -1.23 | -1.12 | -1.67 | -2.68 | -1.04 | -1.09 |
| 17 | 2'-Deoxyadenosine | C10H13N5O3 | 4.28 | 1.05 | -8.36 | -8.40 | -8.46 | -8.50 | -3.64 | -8.82 | -7.32 | -7.89 |
| 18 | Tyramine | C8H11NO | 4.28 | 1.07 | 4.97 | 5.62 | 5.34 | 5.94 | 5.14 | 6.05 | 4.98 | 4.89 |
| 19 | Guanine | C5H5N5O | 4.49 | 1.04 | -4.19 | -3.79 | -3.90 | -3.82 | 4.73 | -3.57 | 1.96 | -3.17 |
| 20 | Guanosine | C10H13N5O5 | 4.49 | 1.11 | -2.60 | -2.20 | -2.55 | -2.47 | 5.96 | -2.60 | 3.67 | -2.22 |
| 21 | Thymine | C5H6N2O2 | 4.85 | 1.10 | 2.60 | 3.66 | 3.51 | 3.02 | 1.84 | 4.07 | 1.92 | 2.96 |
| 22 | 2'-O-Methyladenosine | C11H15N5O4 | 6.66 | 1.07 | -4.84 | -4.82 | -4.78 | -4.76 | 1.54 | -4.81 | -0.98 | -4.82 |
| 23 | Pantothenic acid | C9H17NO5 | 8.66 | 1.09 | -1.32 | -0.86 | -0.87 | -1.11 | -1.16 | -0.37 | -0.71 | -0.71 |
| 24 | PEG n5 | C10H22O6 | 10.46 | 1.28 | 3.08 | 1.92 | 1.33 | 1.88 | 1.26 | 2.98 | 3.91 | 0.20 |
| 25 | 4-Indolecarbaldehyde | C9H7NO | 11.16 | 1.17 | -5.04 | -5.76 | -5.41 | -5.56 | -5.28 | -5.62 | -5.49 | -5.47 |
| 26 | NCGC00179992-02! | C33H40O22 | 11.64 | 1.03 | -0.85 | -0.62 | -0.65 | -0.63 | -0.66 | -1.33 | -0.63 | -0.68 |
| 27 | PEG n6 | C12H26O7 | 11.99 | 1.25 | 5.96 | 4.49 | 3.50 | 4.73 | 3.81 | 5.62 | 6.55 | 2.34 |
| 28 | Hydroxysafflor Yellow A | C27H32O16 | 12.57 | 1.12 | -0.82 | -0.57 | -0.68 | -0.31 | -0.84 | -1.04 | -0.87 | -1.10 |
| 29 | 2-Isopropylmalic acid | C7H12O5 | 12.79 | 1.11 | -0.92 | -0.56 | -0.62 | -1.04 | -1.18 | -0.76 | 0.03 | 1.00 |
| 30 | Chlorogenic acid | C16H18O9 | 13.21 | 1.33 | -1.51 | -0.27 | 0.71 | -0.94 | -0.96 | -4.67 | 2.02 | -0.38 |
| 31 | PEG n7 | C14H30O8 | 13.21 | 1.28 | 6.49 | 5.14 | 3.94 | 5.42 | 4.25 | 6.08 | 7.20 | 2.51 |
| 32 | 2,3,4,9-Tetrahydro-1H-β-carboline-3-carboxylic acid | C12H12N2O2 | 14.14 | 1.06 | 5.31 | 4.63 | 4.85 | 5.23 | 6.05 | 5.61 | 7.15 | 8.13 |
| 33 | PEG n8 | C16H34O9 | 14.32 | 1.27 | 8.46 | 7.08 | 5.87 | 7.37 | 6.41 | 7.96 | 9.10 | 4.49 |
| 34 | 3-Hydroxy-3,5,5-trimethyl-4-(3-oxo-1-buten-1-ylidene)cyclohexyl β-D-glucopyranoside | C19H30O8 | 15.21 | 1.29 | -1.17 | -1.92 | -0.67 | -1.05 | -3.28 | -2.43 | -1.56 | -5.29 |
| 35 | Caffeic acid | C9H8O4 | 15.27 | 1.16 | 1.43 | 2.28 | 2.54 | 5.01 | 1.21 | 3.29 | 4.09 | 4.64 |
| 36 | PEG n10 | C20H42O11 | 16.94 | 1.01 | -2.36 | -4.61 | -7.82 | -7.83 | -7.91 | -7.85 | -3.00 | -6.95 |
| 37 | PEG n11 | C22H46O12 | 17.85 | 1.25 | 10.33 | 9.25 | 8.03 | 9.49 | 8.41 | 9.48 | 11.01 | 6.89 |
| 38 | 5,6,7,8-Tetrahydro-2-naphthol | C10H12O | 18.91 | 1.02 | 1.29 | 2.39 | 2.12 | 2.77 | 1.84 | 1.39 | 1.55 | 2.10 |
| 39 | PEG n12 | C24H50O13 | 18.98 | 1.29 | 9.59 | 8.47 | 7.43 | 8.96 | 7.72 | 9.15 | 10.40 | 6.44 |
| 40 | PEG n13 | C26H54O14 | 20.14 | 1.24 | 8.72 | 7.38 | 6.48 | 8.08 | 6.92 | 8.07 | 9.27 | 5.90 |
| 41 | Quercetin-3β-D-glucoside | C21H20O12 | 21.51 | 1.04 | 3.78 | 4.98 | 5.01 | 2.89 | 4.37 | 1.17 | 4.46 | 4.31 |
| 42 | Trifolin | C21H20O11 | 22.69 | 1.36 | -7.42 | -7.50 | -7.52 | -7.45 | -7.56 | -7.50 | -7.59 | -7.43 |
| 43 | Quercetin | C15H10O7 | 30.75 | 1.03 | 4.45 | 5.78 | 6.44 | 3.54 | 3.33 | -1.28 | 3.18 | 3.66 |
| 44 | Ligustilide | C12H14O2 | 30.95 | 1.01 | -3.04 | -3.15 | -2.63 | -7.42 | -3.12 | -7.09 | -4.85 | -6.99 |
| 45 | Nootkatone | C15H22O | 31.13 | 1.21 | -4.60 | -4.64 | -4.33 | -5.28 | -5.80 | -5.27 | -6.01 | -5.17 |
| 46 | 9S,13R-12-Oxophytodienoic acid | C18H28O3 | 31.24 | 1.03 | 2.40 | 2.80 | 2.21 | 2.10 | 2.48 | 1.92 | 0.13 | -0.03 |
| 47 | (11E,15Z)-9,10,13-trihydroxyoctadeca-11,15-dienoic acid | C18H32O5 | 31.28 | 1.11 | -1.21 | -0.99 | -1.57 | -1.56 | -0.89 | -1.34 | -2.33 | -2.59 |
| 48 | Corchorifatty acid F | C18H32O5 | 31.30 | 1.14 | -0.97 | -0.75 | -1.42 | -1.82 | -0.90 | -1.67 | -2.35 | -2.37 |
| 49 | Genistein | C15H10O5 | 31.81 | 1.18 | 2.42 | 3.86 | 3.45 | 3.12 | 2.91 | 2.53 | 3.85 | 5.38 |
| 50 | (9Z,11E,13S,15Z)-13-hydroxyoctadeca-9,11,15-trienoic acid | C18H30O3 | 31.82 | 1.13 | -1.38 | -1.28 | -1.81 | -1.96 | -1.20 | -1.67 | -2.64 | -2.36 |
| 51 | (15Z)-9,12,13-Trihydroxy-15-octadecenoic acid | C18H34O5 | 31.83 | 1.09 | -1.45 | -1.41 | -1.99 | -2.11 | -1.24 | -1.88 | -2.69 | -2.47 |
| 52 | Pentadecanoic Acid | C15H30O2 | 32.38 | 1.20 | -0.54 | -0.11 | -0.10 | 2.35 | 0.19 | 1.04 | 0.25 | 1.45 |
| 53 | Bis(4-ethylbenzylidene)sorbitol | C24H30O6 | 35.99 | 1.16 | -3.71 | -4.25 | -6.10 | -4.88 | -3.69 | -4.17 | -3.84 | -5.56 |
| 54 | 2-Amino-1,3,4-octadecanetriol | C18H39NO3 | 36.10 | 1.25 | 1.18 | 1.95 | 2.09 | -0.54 | 1.92 | 1.58 | 1.63 | 2.25 |
| 55 | Myristyl sulfate | C14H30O4S | 38.17 | 1.02 | -5.76 | -6.29 | -6.25 | -5.79 | -7.37 | -6.98 | -6.27 | -7.19 |
| 56 | Diisobutylphthalate | C16H22O4 | 38.84 | 1.37 | -3.18 | -1.88 | -1.94 | -5.28 | -2.03 | -1.93 | -1.80 | -1.93 |
| 57 | Dibutyl phthalate | C16H22O4 | 38.95 | 1.02 | 0.85 | -0.49 | -1.99 | 0.93 | 0.66 | 0.76 | 0.90 | 0.76 |
| 58 | Linoleoyl ethanolamide | C20H37NO2 | 40.79 | 1.67 | -1.55 | -0.55 | 3.61 | -1.26 | 2.67 | -0.73 | -0.71 | -0.01 |
| 59 | Bis(2-ethylhexyl) amine | C16H35N | 40.92 | 1.10 | -7.45 | -7.18 | -7.48 | -7.21 | -6.99 | -7.20 | -7.33 | -7.18 |
| 60 | Octadecanamine | C18H39N | 42.19 | 1.79 | 1.73 | 5.60 | 1.51 | 4.04 | 1.54 | 3.58 | 1.42 | 1.90 |
| 61 | 3-[(Carboxycarbonyl)amino]-L-alanine | C5H8N2O5 | 43.07 | 1.12 | 5.87 | 5.18 | 3.79 | 5.70 | 12.52 | 7.91 | 5.19 | 5.17 |
