# Supplementary material for: Effects of drying processes on the chemical and physical properties of safflower: Towards a multidimensional quality evaluation model
Source: PLoS One. 2026 Jan 2;21(1):e0339180. doi: 10.1371/journal.pone.0339180 (PMC12758763; doi:10.1371/journal.pone.0339180)
Supplement: S4 Table — (DOCX) [file pone.0339180.s006.docx]

**S4 Table Relative content of volatile components in safflower samples**

| NO. | Name | Molecular Formula | Molecular weight | RT(Min) | Match | Relative content（%） | | | | | | | | |
| --- | --- | --- | --- | --- | --- | --- | --- | --- | --- | --- | --- | --- | --- | --- |
|  |  |  |  |  |  | **F** | **OD40** | **OD60** | **OD80** | **FD** | **DD** | **SD** | **DFSD** | **NSD** |
| 1 | 2,6-dimethyl-Cyclohexanol | C8H16O | 128.12 | 0.83 | 80 | - | 0.29 | - | - | - | - | - | - | - |
| 2 | m-Cymene | C10H14 | 134.11 | 0.95 | 87 | - | 0.11 | 0.03 | - | - | - | - | - | - |
| 3 | 1,2,3,5-tetramethyl-Benzene | C10H14 | 134.11 | 0.97 | 91 | - | - | - | 0.03 | - | - | - | - | - |
| 4 | p-Cymene | C10H14 | 134.11 | 1.04 | 90 | - | 0.31 | - | - | 0.05 | - | - | - | - |
| 5 | Cyclomethicone 5 | C10H30O5Si5 | 370.09 | 1.48 | 91 | 9.88 | 6.91 | 1.72 | 2.36 | 4.28 | 6.55 | 5.92 | 5.32 | 9.41 |
| 6 | Terpinen-4-ol | C10H18O | 154.14 | 2.31 | 93 | 1.40 | - | 0.03 | - | - | - | - | - | - |
| 7 | Myrtenol | C10H16O | 152.12 | 2.75 | 95 | 4.09 | - | - | - | - | - | - | - | - |
| 8 | 6-methyl-Dodecane | C13H28O | 184.22 | 3.01 | 81 | - | 0.34 | - | - | - | - | - | - | - |
| 9 | 2,6-dimethyl-Undecane | C13H28 | 184.22 | 3.02 | 94 | - | - | 0.15 | 0.15 | - | 0.46 | 0.45 | 0.78 | - |
| 10 | 2-Pinen-4-one | C10H14O | 150.10 | 3.11 | 97 | 1.95 | - | - | 0.13 | 0.12 | 0.40 | 0.31 | - | - |
| 11 | 4-methyl-Dodecane | C13H28 | 184.22 | 3.20 | 96 | - | 0.25 | 0.11 | 0.11 | - | 0.31 | 0.29 | 0.80 | 0.55 |
| 12 | Eucarvone | C10H14O | 150.10 | 3.32 | 83 | - | - | - | - | - | 3.21 | - | - | - |
| 13 | Nonadecane | C19H40 | 268.31 | 3.80 | 81 | - | 0.19 | 0.13 | - | - | - | - | - | - |
| 14 | Eicosane | C20H42 | 282.33 | 3.81 | 87 | - | 0.36 | 0.08 | 0.08 | - | - | - | 0.40 | - |
| 15 | 4,6-dimethyl-Dodecane | C14H30 | 198.24 | 3.93 | 93 | - | - | 0.19 | 0.19 | - | 0.52 | 0.52 | 0.88 | - |
| 16 | 2,6,11-trimethyl-Dodecane | C15H32 | 212.25 | 4.44 | 83 | - | 0.95 | - | - | - | - | 1.00 | 0.52 | - |
| 17 | 3-Ethyl-3-methylheptane | C10H22 | 142.17 | 4.44 | 80 | - | - | - | - | - | 0.87 | - | 1.58 | - |
| 18 | 6-methyl-Tridecane | C14H30 | 198.24 | 4.45 | 80 | - | - | - | - | 0.47 | - | - | - | - |
| 19 | Dodecane | C12H26 | 170.20 | 4.61 | 87 | - | 0.32 | - | - | - | - | - | 0.27 | - |
| 20 | 2,3,6-trimethyl-Decane | C13H28 | 184.22 | 4.62 | 80 | - | - | - | 0.12 | - | 0.43 | 0.49 | 0.64 | - |
| 21 | 2,7,10-trimethyl-Dodecane | C15H32 | 212.25 | 4.72 | 80 | - | 0.26 | - | - | - | - | - | - | - |
| 22 | 5-butyl-Nonane | C13H28 | 184.22 | 4.88 | 90 | - | - | 0.15 | 0.15 | - | - | - | - | - |
| 23 | Oxalic acid, 6-ethyloct-3-yl hexyl ester | C18H34O4 | 314.25 | 5.00 | 86 | - | - | 0.11 | 0.11 | - | 0.34 | - | 0.50 | - |
| 24 | Dodecamethylcyclohexasiloxane | C12H36O6Si6 | 444.11 | 5.36 | 87 | 1.94 | 1.91 | 0.59 | 0.68 | 1.05 | 1.74 | 2.04 | 1.81 | 2.15 |
| 25 | 2-methyl-Tridecane | C14H30 | 198.24 | 5.99 | 94 | - | - | - | 0.10 | 0.08 | - | - | 0.27 | - |
| 26 | 1-Iodoundecane | C11H23I | 282.08 | 5.99 | 94 | - | - | - | - | - | - | 0.26 | - | - |
| 27 | Megastigma-4,6(Z),8(Z)-triene | C13H20 | 176.16 | 6.02 | 80 | 0.14 | - | - | - | - | - | - | - | - |
| 28 | Cyclosativene | C15H24 | 204.19 | 6.16 | 91 | - | 0.48 | 0.37 | 0.36 | 0.25 | 0.37 | - | - | - |
| 29 | .alpha.-Cubebene | C15H24 | 204.19 | 6.29 | 93 | - | 0.52 | 0.63 | 0.55 | 0.25 | 0.25 | - | 0.29 | 0.22 |
| 30 | Tetradecane | C14H30 | 198.24 | 6.61 | 94 | - | 0.41 | 0.39 | 0.37 | 0.23 | 0.70 | 1.12 | 0.72 | 0.52 |
| 31 | (-)-cis-beta-Elemene | C15H24 | 204.19 | 6.55 | 83 | - | - | 0.06 | - | - | - | - | - | - |
| 32 | Cyperene | C15H24 | 204.19 | 6.71 | 95 | - | 3.05 | 2.81 | 2.62 | 2.26 | 1.67 | 2.33 | 2.02 | 2.03 |
| 33 | Nonane | C9H20 | 128.16 | 6.81 | 83 | - | - | - | - | - | - | 0.67 | - | - |
| 34 | Longifolene | C15H24 | 204.19 | 6.81 | 90 | - | - | 0.38 | 0.38 | - | - | - | - | - |
| 35 | Isolongifolene, 9,10-dehydro- | C15H22 | 202.17 | 6.93 | 83 | - | 1.35 | - | - | - | - | - | - | - |
| 36 | Caryophyllene | C15H24 | 204.19 | 7.04 | 99 | 12.41 | 31.12 | 49.90 | 51.38 | 40.10 | 16.98 | 21.02 | 22.16 | 23.75 |
| 37 | (+)-2-Carene | C10H16 | 136.13 | 7.15 | 83 | - | - | - | - | - | - | - | 0.43 | - |
| 38 | 10,10-Dimethyl-2,6-dimethylenebicyclo[7.2.0]undecane | C15H24 | 204.19 | 7.15 | 91 | - | - | - | 0.36 | - | - | - | - | - |
| 39 | Tridecane | C13H28 | 184.22 | 7.21 | 93 | - | - | - | - | - | 0.22 | 0.27 | - | - |
| 40 | Sulfurous acid, butyl decyl ester | C14H30O3S | 278.19 | 7.21 | 80 | - | - | - | - | 0.12 | - | - | - | - |
| 41 | 1,1'-oxybis-Decane | C20H42O | 298.32 | 7.37 | 80 | - | - | - | - | - | 0.29 | 0.31 | - | - |
| 42 | 3,8-dimethyl-Decane | C12H26 | 170.20 | 7.42 | 83 | - | - | - | 0.16 | - | - | - | - | - |
| 43 | Alloaromadendrene | C15H24 | 204.19 | 7.48 | 94 | - | - | 0.14 | 0.14 | - | - | - | - | - |
| 44 | Humulene | C15H24 | 204.19 | 7.54 | 95 | - | 1.83 | 2.69 | 2.84 | 2.37 | 1.63 | 1.76 | 2.05 | 1.94 |
| 45 | Dimethyl phthalate | C10H10O4 | 194.06 | 7.59 | 94 | 4.97 | - | - | - | - | - | - | - | - |
| 46 | 2,4-dimethyl-Heptane | C9H20 | 128.16 | 7.60 | 80 | - | 0.21 | - | - | - | - | - | - | - |
| 47 | rotundene | C15H24 | 204.19 | 7.63 | 98 | - | - | - | 0.27 | - | - | - | - | - |
| 48 | (+)-epi-Bicyclosesquiphellandrene | C15H24 | 204.19 | 7.68 | 96 | - | - | 0.49 | - | - | - | - | - | - |
| 49 | 3-methyl-Tetradecane | C15H32 | 212.25 | 7.69 | 96 | - | - | - | 0.13 | - | - | - | - | - |
| 50 | (+)-Gamma-cadinene | C15H24 | 204.19 | 7.85 | 86 | - | - | 0.31 | 0.29 | - | - | - | - | - |
| 51 | Selina-3,7(11)-diene | C15H24 | 204.19 | 7.89 | 93 | - | - | 0.27 | - | - | - | - | - | - |
| 52 | beta-Cubebene | C15H24 | 204.19 | 7.95 | 97 | - | - | 0.45 | - | - | - | - | - | - |
| 53 | trans-.beta.-Ionone | C13H20O | 192.15 | 8.00 | 97 | 2.73 | - | - | - | - | - | - | - | - |
| 54 | 1-Pentadecene | C15H30 | 210.24 | 8.01 | 99 | - | - | 4.94 | - | - | - | - | - | - |
| 55 | 1-Tridecene | C13H26 | 182.20 | 8.01 | 95 | - | 4.21 | - | 5.16 | 3.93 | - | - | - | - |
| 56 | Pentadecane | C15H32 | 212.25 | 8.11 | 97 | - | - | 0.79 | 0.85 | 0.29 | 0.32 | - | 0.93 | - |
| 57 | 3-methyl-1,1'-Biphenyl | C13H12 | 168.09 | 8.32 | 91 | - | - | - | - | 0.27 | - | - | - | - |
| 58 | alpha-Bulnesene | C15H24 | 204.19 | 8.23 | 83 | - | - | 1.01 | - | - | - | - | - | - |
| 59 | 2,4-bis(1,1-dimethylethyl)-Phenol | C14H22O | 206.17 | 8.40 | 80 | 0.64 | 0.55 | - | - | 0.62 | - | 0.66 | 0.53 | 0.58 |
| 60 | Cadina-1(10),4-diene | C15H24 | 204.19 | 8.56 | 83 | 0.55 | 0.74 | 1.59 | 1.57 | 1.03 | 0.90 | 1.56 | 1.08 | 1.10 |
| 61 | (+/-)-dihydroactinidiolide | C11H16O2 | 180.12 | 8.76 | 97 | - | 5.11 | 1.57 | 1.56 | 2.12 | 6.33 | 6.06 | 5.62 | 6.64 |
| 62 | (-)-Isolongifolol | C15H26O | 222.20 | 9.08 | 84 | - | - | 0.33 | - | - | - | 0.60 | - | - |
| 63 | Caryophyllene oxide | C15H24O | 220.18 | 9.64 | 94 | - | 6.27 | 1.83 | 1.75 | 3.46 | 6.50 | 5.27 | 5.02 | 5.45 |
| 64 | Propanoic acid, 2-methyl-, 1-(1,1-dimethylethyl)-2-methyl-1,3-propanediyl ester | C16H30O4 | 286.21 | 9.80 | 80 | - | - | - | 0.51 | - | - | - | - | - |
| 65 | 3,5-Dimethylcyclohex-1-ene-4-carboxaldehyde | C9H14O | 138.10 | 10.16 | 80 | - | 0.14 | - | - | - | - | - | - | - |
| 66 | alpha-Caryophylladienol | C15H24O | 220.18 | 10.75 | 90 | - | - | - | 1.36 | 2.29 | 1.75 | - | 0.95 | 0.99 |
| 67 | 2-Naphthalenemethanol, decahydro-.alpha.,.alpha.,4a-trimethyl-8-methylene-, [2R-(2.alpha.,4a.alpha.,8a.beta.)]- | C15H26O | 222.20 | 11.06 | 90 | - | 0.12 | - | - | - | 0.23 | - | - | - |
| 68 | 1-Methyl-6-methylenebicyclo[3.2.0]heptane | C9H14 | 122.11 | 11.22 | 80 | - | - | - | - | - | - | 0.69 | - | - |
| 69 | (Z,Z,Z)-9,12,15-Octadecatrien-1-ol | C18H32O | 264.25 | 11.32 | 83 | - | - | - | - | - | - | 0.48 | - | - |
| 70 | 1,4-Cyclooctadiene, (Z,Z)- | C8H12 | 108.09 | 11.32 | 87 | - | - | - | - | - | 0.46 | - | - | - |
| 71 | 3-Dodecen-1-yne, (E)- | C12H20 | 164.16 | 11.43 | 81 | - | - | - | - | - | - | - | - | 0.18 |
| 73 | 4-Allyltoluene | C10H12 | 132.09 | 15.59 | 87 | - | - | 3.71 | 4.33 | 7.45 | 8.35 | - | - | - |
| 72 | Hexahydrofarnesylacetone | C18H36O | 268.28 | 16.32 | 81 | - | 0.78 | - | - | - | - | 1.20 | 0.79 | - |
| 74 | Hexadecanoic acid, ethyl ester | C18H36O2 | 284.27 | 19.96 | 91 | 0.37 | - | - | - | - | - | - | - | - |
| 75 | Tricosane | C23H48 | 324.38 | 25.88 | 87 | - | - | - | 0.03 | - | - | - | - | - |
| 76 | Supraene | C30H50 | 410.39 | 34.07 | 99 | - | 1.67 | - | 0.06 | - | - | - | - | - |

“-” represent not detected.
