# Supplementary material for: Effects of drying processes on the chemical and physical properties of safflower: Towards a multidimensional quality evaluation model
Source: PLoS One. 2026 Jan 2;21(1):e0339180. doi: 10.1371/journal.pone.0339180 (PMC12758763; doi:10.1371/journal.pone.0339180)
Supplement: S7 Table — (DOCX) [file pone.0339180.s009.docx]

**S7 Table** Machine learning sample information

| **S** | **Q** | **N** | **M** | **T (℃)** | **L*** | **a*** | **b*** | **ΔE*** | **R** | **G** | **B** | **LD** | **Content (μg/g)** | | | | | | | |
| --- | --- | --- | --- | --- | --- | --- | --- | --- | --- | --- | --- | --- | --- | --- | --- | --- | --- | --- | --- | --- |
|  |  |  |  |  |  |  |  |  |  |  |  |  | **HSYA** | **AB** | **HKT** | **KD** | **HKR** | **HKD** | **HAG** | **KR** |
| S1 | 2 | 0 | 1 | -40 | 54.76 | 26.31 | 29.04 | 63.51 | 225 | 108 | 29 | 135.5 | 36831.81 | 26221.88 | 2553.93 | 248.86 | 338.03 | / | 655.95 | / |
| S2 | 1 | 0 | 2 | 25 | 48.02 | 22.63 | 19.19 | 0.79 | 203 | 86 | 35 | 115.9 | 23939.92 | 20910.93 | 3552.28 | 330.51 | 718.22 | / | 748.54 | / |
| S3 | 1 | 0 | 3 | 15 | 49.05 | 24.22 | 21.14 | 1.12 | 228 | 108 | 46 | 137.4 | 30120.39 | 22929.87 | 2751.43 | 523.79 | 479.41 | / | 663.32 | / |
| S4 | 1 | 0 | 4 | 30 | 49.49 | 25.27 | 20.97 | 2.94 | 214 | 96 | 36 | 125.3 | 23601.60 | 18533.18 | 3425.38 | 246.07 | 462.21 | / | 689.93 | / |
| S5 | 1 | 0 | 5 | 30 | 47.97 | 24.49 | 19.28 | 1.41 | 221 | 94 | 36 | 126.2 | 29034.20 | 25318.44 | 2641.95 | 258.84 | 933.17 | 366.40 | 769.21 | 2401.00 |
| S6 | 2 | 0 | 6 | 40 | 49.40 | 24.87 | 21.08 | 2.20 | 221 | 95 | 35 | 126.7 | 32769.93 | 24087.48 | 2687.31 | 313.98 | 360.10 | / | 735.74 | 1712.30 |
| S7 | 3 | 0 | 6 | 60 | 45.68 | 22.86 | 16.77 | 9.92 | 217 | 197 | 30 | 186.4 | 33243.36 | 23394.87 | 2706.3 | 456.84 | 562.62 | 317.12 | 673.17 | 1933.96 |
| S8 | 4 | 0 | 6 | 80 | 43.57 | 14.86 | 14.20 | 64.86 | 217 | 96 | 29 | 125.8 | 32182.24 | 21571.56 | 2874.05 | 539.05 | 1106.96 | 491.11 | 610.36 | 2273.33 |
| S9 | 2 | 0 | 1 | -40 | 54.56 | 26.33 | 28.86 | 60.76 | 230 | 113 | 33 | 140.1 | 36847.52 | 26284.62 | 2624.94 | 269.11 | / | / | 661.69 | / |
| S10 | 1 | 0 | 2 | 25 | 47.83 | 22.58 | 19.02 | 1.11 | 213 | 94 | 45 | 124.8 | 24019.04 | 21581.91 | 2692.15 | 393.53 | 1187.58 | / | 822.74 | / |
| S11 | 1 | 0 | 3 | 15 | 48.93 | 24.27 | 21.12 | 1.09 | 229 | 110 | 47 | 139.4 | 30139.76 | 23065.63 | 2743.18 | 524.40 | 587.25 | / | 663.98 | / |
| S12 | 1 | 0 | 4 | 30 | 49.29 | 25.26 | 20.90 | 2.70 | 220 | 101 | 40 | 130.6 | 23606.15 | 18518.46 | 3420.35 | 3728.12 | 456.40 | / | 691.74 | / |
| S13 | 1 | 0 | 5 | 30 | 47.95 | 24.46 | 19.23 | 1.43 | 218 | 93 | 33 | 124.5 | 29009.46 | 24983.98 | 2652.42 | 262.96 | 842.99 | 374.84 | 763.55 | 2466.03 |
| S14 | 2 | 0 | 6 | 40 | 49.37 | 24.91 | 21.07 | 2.24 | 224 | 95 | 36 | 127.8 | 32725.06 | 23842.84 | 2692.87 | 316.76 | 395.42 | / | 734.11 | 1741.42 |
| S15 | 3 | 0 | 6 | 60 | 44.33 | 19.65 | 14.36 | 32.03 | 219 | 400 | 32 | 308.9 | 33250.41 | 22886.61 | 2760.30 | 579.11 | 610.63 | 323.55 | 670.12 | 2025.44 |
| S16 | 4 | 0 | 6 | 80 | 45.30 | 22.71 | 16.47 | 12.20 | 215 | 97 | 29 | 125.6 | 32187.25 | 21538.14 | 2909.67 | 609.82 | 865.80 | 422.89 | 611.58 | 2313.50 |
| S17 | 2 | 0 | 1 | -40 | 54.83 | 26.33 | 29.14 | 64.90 | 219 | 101 | 24 | 128.7 | 36836.65 | 26491.83 | 2602.43 | 267.27 | / | / | 655.00 | / |
| S18 | 1 | 0 | 2 | 25 | 48.00 | 22.66 | 19.21 | 0.76 | 197 | 80 | 27 | 109.8 | 24003.97 | 20565.25 | 3862.08 | 359.12 | 528.12 | / | 710.57 | / |
| S19 | 1 | 0 | 3 | 15 | 49.16 | 24.20 | 21.26 | 1.29 | 225 | 108 | 48 | 137.1 | 30063.57 | 22679.64 | 2741.74 | 519.43 | 429.59 | / | 661.13 | / |
| S20 | 1 | 0 | 4 | 30 | 49.58 | 25.26 | 20.96 | 3.02 | 213 | 95 | 37 | 124.6 | 23597.05 | 18547.90 | 3430.42 | 3733.92 | 468.01 | / | 688.12 | / |
| S21 | 1 | 0 | 5 | 30 | 47.95 | 24.49 | 19.30 | 1.40 | 217 | 92 | 36 | 123.9 | 28940.14 | 24992.87 | 2645.87 | 264.26 | 850.21 | 384.1 | 763.80 | 2463.65 |
| S22 | 2 | 0 | 6 | 40 | 49.40 | 24.85 | 21.04 | 2.14 | 221 | 94 | 34 | 126.1 | 32737.73 | 23875.93 | 2712.81 | 318.89 | 390.82 | / | 736.60 | 1714.72 |
| S23 | 3 | 0 | 6 | 60 | 44.33 | 19.73 | 14.35 | 31.81 | 216 | 96 | 28 | 125.2 | 33187.00 | 23607.49 | 2703.11 | 403.99 | 596.93 | 310.70 | 670.24 | 2012.07 |
| S24 | 4 | 0 | 6 | 80 | 45.89 | 22.96 | 16.93 | 8.79 | 218 | 95 | 30 | 125.4 | 32158.38 | 21529.47 | 2888.50 | 582.24 | 858.58 | 416.41 | 608.02 | 2330.10 |
| S25 | 1 | 4 | 2 | 25 | 50.29 | 21.23 | 23.55 | 14.05 | 201 | 93 | 26 | 118.7 | 23387.20 | 6632.20 | 11294.25 | 1419.30 | 3825.95 | 5008.80 | 860.40 | 474.50 |
| S26 | 1 | 3 | 2 | 25 | 46.21 | 22.26 | 18.07 | 14.44 | 203 | 92 | 36 | 119.7 | 18162.65 | 3328.75 | 5595.85 | / | 1214.30 | 2196.30 | 441.30 | / |
| S27 | 2 | 3 | 4 | 45 | 52.45 | 28.22 | 24.28 | 28.01 | 224 | 105 | 39 | 134.1 | 22315.15 | 2318.70 | 5946.10 | 302.75 | 1616.25 | 1793.15 | 532.50 | 686.65 |
| S28 | 2 | 3 | 4 | 45 | 53.32 | 27.77 | 27.55 | 50.46 | 225 | 106 | 37 | 134.8 | 22586.35 | 1990.85 | 6787.50 | 427.30 | 1333.90 | 2006.25 | 428.90 | / |
| S29 | 2 | 3 | 4 | 45 | 54.33 | 28.71 | 25.72 | 43.16 | 220 | 100 | 33 | 129.3 | 26698.60 | 2398.50 | 6006.85 | 315.55 | 1805.7 | 1608.15 | 1172.35 | / |
| S30 | 1 | 3 | 4 | 45 | 53.25 | 28.73 | 25.40 | 38.61 | 211 | 96 | 35 | 124.4 | 26826.60 | 5798.65 | 9104.30 | 284.95 | 2327.05 | 4782.45 | 1563.10 | 666.80 |
| S31 | 1 | 3 | 4 | 45 | 49.02 | 24.96 | 21.41 | 7.90 | 205 | 93 | 33 | 120.6 | 17062.75 | 3424.35 | 6067.50 | 327.65 | 1848.65 | 5670.50 | 1759.85 | / |
| S32 | 2 | 3 | 2 | 25 | 53.73 | 24.39 | 27.38 | 40.23 | 223 | 106 | 26 | 133.1 | 21625.55 | 2350.45 | 3557.85 | / | 969.90 | 866.85 | 621.80 | 639.55 |
| S33 | 2 | 3 | 4 | 45 | 53.15 | 27.75 | 27.02 | 45.59 | 214 | 97 | 27 | 125.1 | 25893.25 | 4094.25 | 6033.85 | / | 1581.80 | 2648.15 | 1303.95 | 620.00 |
| S34 | 2 | 3 | 4 | 45 | 50.68 | 20.90 | 25.35 | 24.64 | 216 | 107 | 33 | 132.3 | 19727.95 | 3992.55 | 4068.85 | 452.40 | 1744.95 | 3918.20 | / | 543.30 |
| S35 | 4 | 2 | 4 | 45 | 51.51 | 19.97 | 23.77 | 17.77 | 156 | 62 | 6 | 84.6 | 15048.7 | 2945.85 | 5465.65 | 336.60 | 1117.40 | 1905.70 | 682.35 | 854.0 |
| S36 | 4 | 2 | 4 | 45 | 52.41 | 21.69 | 24.95 | 20.71 | 164 | 64 | 5 | 88.1 | 16665.85 | 3788.95 | 6167.70 | 371.65 | 1771.65 | 2678.65 | 800.05 | 661.55 |
| S37 | 4 | 2 | 4 | 45 | 51.67 | 21.06 | 23.57 | 13.87 | 176 | 73 | 7 | 97.3 | 15325.15 | 2698.30 | 3965.70 | / | 1223.75 | 2027.00 | 552.35 | 459.15 |
| S38 | 4 | 2 | 4 | 35 | 49.99 | 19.74 | 14.30 | 17.22 | 155 | 62 | 8 | 84.5 | 14742.40 | 3550.00 | 5300.40 | / | 1228.15 | 2262.45 | 491.65 | 489.15 |
| S39 | 4 | 2 | 4 | 45 | 52.86 | 20.65 | 17.18 | 5.62 | 163 | 68 | 9 | 90.6 | 14704.40 | 3401.40 | 5156.25 | / | 1226.35 | 2398.20 | 800.75 | 962.95 |
| S40 | 4 | 2 | 4 | 45 | 51.83 | 20.54 | 16.47 | 6.38 | 168 | 70 | 5 | 92.9 | 15140.10 | 3301.60 | 5315.75 | / | 1219.75 | 2565.60 | 902.45 | 908.30 |
| S41 | 4 | 2 | 4 | 45 | 52.72 | 20.49 | 17.41 | 5.52 | 167 | 72 | 3 | 93.6 | 15488.30 | 35320.00 | 5723.10 | / | 1335.10 | 2318.65 | 865.65 | 977.75 |
| S42 | 4 | 2 | 4 | 45 | 53.23 | 20.34 | 17.39 | 6.77 | 164 | 71 | 8 | 92.6 | 14214.30 | 3459.30 | 5565.80 | / | 1318.90 | 2352.75 | 720.90 | 967.55 |
| S43 | 4 | 2 | 4 | 45 | 52.94 | 20.76 | 17.71 | 4.77 | 182 | 82 | 8 | 104.6 | 16013.25 | 3297.20 | 5742.30 | / | 1357.05 | 2362.20 | 865.90 | 919.15 |
| S44 | 2 | 2 | 4 | 45 | 57.02 | 20.73 | 20.40 | 19.95 | 225 | 122 | 39 | 144.6 | 16480.65 | 3410.45 | 4842.65 | 677.90 | 1529.85 | 1996.95 | 647.25 | 400.50 |
| S45 | 2 | 2 | 4 | 45 | 53.61 | 24.36 | 17.74 | 3.50 | 220 | 108 | 38 | 134.6 | 22376.50 | 3422.05 | 7090.10 | 1951.20 | 1546.70 | 3050.60 | 522.25 | 812.55 |
| S46 | 3 | 2 | 4 | 35 | 56.34 | 21.74 | 19.03 | 13.10 | 238 | 130 | 57 | 155.1 | 12888.90 | 2040.75 | 2826.50 | 676.50 | 946.05 | 1320.05 | / | 255.10 |
| S47 | 2 | 2 | 2 | 25 | 49.64 | 24.38 | 14.36 | 12.06 | 203 | 90 | 33 | 118.2 | 15551.05 | 5014.55 | 9550.40 | 1100.25 | 1268.10 | 5052.55 | 792.10 | 1434.45 |
| S48 | 1 | 2 | 2 | 25 | 49.86 | 24.06 | 14.68 | 10.02 | 218 | 101 | 37 | 129.7 | 15628.75 | 3604.65 | 5788.25 | 999.30 | 2121.55 | 6003.55 | 1966.60 | 423.80 |
| S49 | 4 | 2 | 2 | 25 | 50.93 | 19.22 | 15.22 | 14.58 | 203 | 101 | 31 | 124.6 | 7479.00 | 1359.45 | 6164.25 | 1862.25 | 1017.20 | 7810.55 | 761.35 | 407.20 |
| S50 | 3 | 1 | 2 | 25 | 47.32 | 21.64 | 12.20 | 31.42 | 220 | 108 | 48 | 135.6 | 24999.85 | 9430.80 | 14041.55 | 910.55 | 4503.25 | 6954.80 | 821.50 | 1275.45 |
| S51 | 1 | 1 | 4 | 35 | 51.61 | 25.54 | 16.42 | 5.45 | 222 | 106 | 39 | 134.1 | 27442.00 | 5111.15 | 7496.45 | 578.90 | 1858.65 | 3744.90 | 349.45 | 565.80 |
| S52 | 2 | 1 | 4 | 35 | 51.65 | 25.68 | 16.52 | 5.54 | 211 | 96 | 30 | 123.9 | 27422.95 | 4712.55 | 6660.45 | 352.55 | 1993.55 | 3772.75 | 30.50 | 693.20 |
| S53 | 2 | 1 | 4 | 35 | 47.23 | 21.94 | 12.58 | 28.98 | 195 | 95 | 29 | 118.4 | 15058.10 | 4396.00 | 6036.30 | 362.25 | 2752.75 | 6935.85 | 198.05 | 1015.25 |
| S54 | 3 | 1 | 4 | 30 | 49.14 | 20.13 | 13.59 | 20.94 | 208 | 97 | 38 | 124.4 | 18753.85 | 2813.35 | 5212.40 | / | 1237.05 | 2057.25 | 525.40 | / |
| S55 | 2 | 1 | 4 | 30 | 52.97 | 23.55 | 18.22 | 1.36 | 231 | 115 | 42 | 142.5 | 29444.35 | 8101.05 | 9600.85 | 319.35 | 3207.55 | 3700.05 | 1444.00 | 1408.30 |
| S56 | 1 | 1 | 4 | 30 | 49.38 | 23.81 | 14.47 | 11.59 | 218 | 106 | 43 | 133.3 | 24684.45 | 8059.10 | 7893.40 | 329.20 | 2379.20 | 3126.45 | 1065.90 | 767.05 |
| S57 | 3 | 1 | 4 | 30 | 49.94 | 23.14 | 15.19 | 7.59 | 202 | 91 | 29 | 118.1 | 25884.40 | 4617.10 | 6191.50 | / | 2130.25 | 2528.05 | 850.60 | 369.95 |
| S58 | 1 | 1 | 4 | 45 | 49.07 | 24.26 | 14.51 | 12.48 | 214 | 95 | 33 | 124.5 | 27277.70 | 5067.50 | 6362.00 | / | 1874.70 | 2457.20 | 857.30 | 643.65 |
| S59 | 2 | 1 | 4 | 45 | 50.36 | 23.49 | 15.72 | 5.30 | 200 | 84 | 23 | 112.7 | 25131.85 | 5379.70 | 5628.65 | / | 1594.20 | 1897.55 | 920.05 | 399.50 |
| S60 | 3 | 1 | 4 | 45 | 50.74 | 24.80 | 16.53 | 4.00 | 225 | 108 | 38 | 136.1 | 27287.45 | 5748.20 | 6433.95 | 448.85 | 1666.95 | 2451.30 | 912.40 | 580.75 |
| S61 | 1 | 1 | 4 | 45 | 55.15 | 24.29 | 18.91 | 7.39 | 207 | 108 | 32 | 130.1 | 20819.55 | 2032.35 | 5928.30 | 1295.25 | 1268.05 | 2110.60 | 331.60 | 564.10 |
| S62 | 3 | 1 | 4 | 45 | 50.33 | 24.28 | 15.62 | 6.18 | 221 | 114 | 43 | 139.0 | 22600.95 | 3736.30 | 5047.15 | 417.35 | 1495.65 | 2162.25 | 769.80 | 271.45 |
| S63 | 1 | 1 | 4 | 45 | 50.61 | 24.43 | 15.78 | 5.57 | 214 | 98 | 39 | 126.9 | 23784.60 | 3737.95 | 5076.00 | 305.80 | 1483.25 | 1980.30 | 837.70 | 343.45 |
| S64 | 1 | 1 | 4 | 45 | 49.68 | 24.13 | 14.91 | 9.46 | 224 | 106 | 40 | 134.8 | 22932.75 | 4039.20 | 5218.75 | 296.10 | 1410.15 | 2004.05 | 821.50 | / |
| S65 | 1 | 4 | 2 | 25 | 50.47 | 21.27 | 22.90 | 10.91 | 201 | 93 | 26 | 118.7 | 23413.19 | 6945.73 | 11303.05 | 1296.17 | 3525.06 | 4828.19 | 819.43 | 410.63 |
| S66 | 1 | 3 | 2 | 25 | 49.91 | 21.15 | 22.29 | 9.53 | 203 | 92 | 36 | 118.7 | 18132.20 | 3408.98 | 5581.81 | / | 1443.74 | 2266.76 | 357.68 | / |
| S67 | 2 | 3 | 4 | 45 | 46.20 | 22.34 | 17.59 | 14.89 | 224 | 105 | 39 | 119.7 | 22279.68 | 2309.53 | 5920.55 | 297.80 | 1621.06 | 1792.32 | 501.50 | 671.43 |
| S68 | 2 | 3 | 4 | 45 | 46.22 | 22.14 | 17.42 | 15.21 | 225 | 106 | 37 | 119.7 | 22543.64 | 1988.81 | 6782.05 | 323.35 | 1337.41 | 2006.29 | 417.90 | / |
| S69 | 2 | 3 | 4 | 45 | 52.35 | 28.19 | 26.08 | 39.30 | 220 | 100 | 33 | 134.1 | 26729.64 | 2395.88 | 6002.00 | 314.77 | 1800.96 | 1605.85 | 1178.27 | / |
| S70 | 1 | 3 | 4 | 45 | 52.17 | 28.29 | 25.93 | 38.57 | 211 | 96 | 35 | 134.1 | 26834.34 | 5744.60 | 9108.69 | 281.59 | 2336.73 | 4765.33 | 1553.44 | 653.19 |
| S71 | 1 | 3 | 4 | 45 | 53.33 | 27.76 | 27.44 | 49.48 | 205 | 93 | 33 | 134.8 | 17072.21 | 3432.96 | 6101.12 | 325.05 | 1845.12 | 5671.30 | 1728.76 | / |
| S72 | 2 | 3 | 2 | 25 | 53.15 | 27.70 | 27.31 | 47.77 | 223 | 106 | 26 | 134.8 | 21638.41 | 2352.13 | 3545.53 | / | 989.08 | 905.80 | 622.19 | 706.24 |
| S73 | 2 | 3 | 4 | 45 | 54.09 | 28.69 | 27.58 | 57.06 | 214 | 97 | 27 | 129.3 | 25844.46 | 3811.71 | 6056.25 | / | 1736.33 | 3077.90 | / | 616.29 |
| S74 | 2 | 3 | 4 | 45 | 54.32 | 28.78 | 27.73 | 59.52 | 216 | 107 | 33 | 129.3 | 19718.38 | 4022.01 | 4060.98 | 456.84 | 1749.15 | 3903.90 | 1303.47 | 541.04 |
| S75 | 4 | 2 | 4 | 45 | 53.18 | 28.78 | 26.66 | 47.89 | 156 | 62 | 6 | 124.4 | 15032.75 | 2955.51 | 5485.62 | / | 1188.23 | 1980.90 | 558.47 | 856.26 |
| S76 | 4 | 2 | 4 | 45 | 53.56 | 28.78 | 26.90 | 50.54 | 164 | 64 | 5 | 124.4 | 16657.00 | 3793.35 | 6166.46 | 367.86 | 1768.87 | 2698.91 | 818.88 | 675.42 |
| S77 | 4 | 2 | 4 | 45 | 49.04 | 25.05 | 21.17 | 7.40 | 176 | 73 | 7 | 120.6 | 15332.49 | 2677.02 | 3967.94 | / | 925.78 | 1710.39 | 346.71 | 455.04 |
| S78 | 4 | 2 | 4 | 35 | 49.04 | 25.00 | 21.10 | 7.14 | 155 | 62 | 8 | 120.6 | 14722.48 | 3555.82 | 5345.18 | / | 1225.63 | 2224.47 | 358.15 | 487.78 |
| S79 | 4 | 2 | 4 | 45 | 53.71 | 24.43 | 29.14 | 56.93 | 163 | 68 | 9 | 133.1 | 14710.34 | 3398.19 | 5162.45 | / | 1250.83 | 2490.34 | 860.82 | 959.77 |
| S80 | 4 | 2 | 4 | 45 | 53.84 | 24.43 | 29.23 | 58.17 | 168 | 70 | 5 | 133.1 | 15133.63 | 3297.41 | 5318.90 | / | 1220.35 | 2558.35 | 902.62 | 907.05 |
| S81 | 4 | 2 | 4 | 45 | 53.00 | 27.70 | 27.97 | 53.38 | 167 | 72 | 3 | 125.1 | 15494.03 | 3538.81 | 5594.02 | / | 1326.39 | 2316.97 | 863.76 | 976.38 |
| S82 | 4 | 2 | 4 | 45 | 53.14 | 27.80 | 28.18 | 56.00 | 164 | 71 | 8 | 125.1 | 14218.26 | 3456.97 | 5556.67 | / | 1312.56 | 2350.99 | 805.64 | 966.48 |
| S83 | 4 | 2 | 4 | 45 | 50.86 | 20.94 | 23.87 | 15.77 | 182 | 82 | 8 | 132.3 | 16006.96 | 3296.64 | 5733.41 | / | 1358.28 | 2375.51 | 871.03 | 920.86 |
| S84 | 2 | 2 | 4 | 45 | 50.50 | 20.88 | 23.57 | 14.71 | 225 | 122 | 39 | 132.3 | 16493.54 | 3440.46 | 4834.84 | 773.32 | 1560.61 | 2179.37 | 898.97 | 598.01 |
| S85 | 2 | 2 | 4 | 45 | 51.68 | 20.01 | 23.95 | 18.61 | 220 | 108 | 38 | 84.6 | 22388.68 | 3382.62 | 7145.91 | 1935.37 | 1579.77 | 3017.20 | 534.92 | 817.21 |
| S86 | 3 | 2 | 4 | 35 | 51.27 | 19.89 | 23.80 | 18.23 | 238 | 130 | 57 | 84.6 | 12877.72 | 2153.63 | 2838.71 | 670.38 | 949.47 | 1367.47 | / | 252.33 |
| S87 | 2 | 2 | 2 | 25 | 52.16 | 21.69 | 25.50 | 24.03 | 203 | 90 | 33 | 88.1 | 15609.03 | 5025.94 | 9551.16 | 1087.57 | 1261.38 | 5014.15 | 797.14 | 1452.90 |
| S88 | 1 | 2 | 2 | 25 | 52.70 | 21.72 | 25.84 | 26.86 | 218 | 101 | 37 | 88.1 | 15629.29 | 3614.05 | 5766.67 | 1180.74 | 2117.72 | 5952.78 | 1925.00 | 429.61 |
| S89 | 4 | 2 | 2 | 25 | 51.83 | 21.07 | 24.45 | 18.50 | 203 | 101 | 31 | 97.3 | 7476.60 | 1364.11 | 6139.03 | 1890.83 | 1015.20 | 8071.47 | 982.36 | 405.36 |
| S90 | 3 | 1 | 2 | 25 | 51.95 | 21.12 | 24.47 | 18.56 | 220 | 108 | 48 | 97.3 | 25008.17 | 9471.18 | 14047.66 | 908.17 | 4504.99 | 6937.11 | 842.03 | 1297.48 |
| S91 | 1 | 1 | 4 | 35 | 49.78 | 19.76 | 21.45 | 11.01 | 222 | 106 | 39 | 84.5 | 27534.24 | 5128.73 | 7536.29 | 580.71 | 1867.41 | 3772.18 | 354.21 | 570.30 |
| S92 | 2 | 1 | 4 | 35 | 50.20 | 19.85 | 21.67 | 10.70 | 211 | 96 | 30 | 84.5 | 27447.88 | 4713.96 | 6642.84 | 354.66 | 1998.32 | 3786.34 | 29.70 | 667.58 |
| S93 | 2 | 1 | 4 | 35 | 52.86 | 20.65 | 25.74 | 28.57 | 195 | 95 | 29 | 90.6 | 15063.77 | 4399.06 | 6045.92 | 361.19 | 2768.79 | 6904.96 | 197.93 | 1013.97 |
| S94 | 3 | 1 | 4 | 30 | 52.83 | 20.67 | 25.78 | 28.75 | 208 | 97 | 38 | 90.6 | 18728.51 | 2814.54 | 5219.73 | / | 1427.10 | 2286.76 | 693.34 | / |
| S95 | 2 | 1 | 4 | 30 | 51.95 | 20.55 | 24.69 | 21.21 | 231 | 115 | 42 | 92.9 | 29424.56 | 8095.83 | 9587.22 | 318.10 | 3200.16 | 3694.4 | 1479.05 | 1461.34 |
| S96 | 1 | 1 | 4 | 30 | 51.59 | 20.55 | 24.38 | 19.31 | 218 | 106 | 43 | 92.9 | 24687.78 | 7914.18 | 7922.27 | / | 2407.98 | 3168.75 | 1024.46 | 763.40 |
| S97 | 3 | 1 | 4 | 30 | 52.74 | 20.49 | 26.08 | 31.26 | 202 | 91 | 29 | 93.6 | 25926.37 | 4673.50 | 6426.34 | / | 1836.36 | 2113.67 | 754.21 | 370.66 |
| S98 | 1 | 1 | 4 | 45 | 52.69 | 20.51 | 26.11 | 31.36 | 214 | 95 | 33 | 93.6 | 27284.62 | 4269.24 | 6799.16 | / | 1869.16 | 2448.02 | 860.25 | 660.83 |
| S99 | 2 | 1 | 4 | 45 | 53.23 | 20.33 | 26.07 | 32.39 | 200 | 84 | 23 | 92.6 | 25136.92 | 5417.24 | 5622.35 | / | 1710.10 | 2095.07 | 946.37 | 410.99 |
| S100 | 3 | 1 | 4 | 45 | 53.24 | 20.34 | 26.03 | 32.09 | 225 | 108 | 38 | 92.6 | 27291.86 | 5757.57 | 5810.11 | 450.81 | 1661.41 | 2524.60 | 915.89 | 596.11 |
| S101 | 1 | 1 | 4 | 45 | 52.94 | 20.77 | 26.58 | 34.58 | 207 | 108 | 32 | 104.6 | 20792.47 | 2021.83 | 6031.03 | 1291.73 | 1267.85 | 2090.80 | 335.16 | 562.56 |
| S102 | 3 | 1 | 4 | 45 | 52.93 | 20.75 | 26.57 | 34.54 | 221 | 114 | 43 | 104.6 | 22599.21 | 3737.41 | 5024.09 | 414.44 | 1491.54 | 2173.31 | 578.76 | 270.57 |
| S103 | 1 | 1 | 4 | 45 | 57.10 | 20.69 | 30.70 | 90.26 | 214 | 98 | 39 | 144.6 | 23771.69 | 3739.83 | 5118.02 | 308.53 | 1482.26 | 1977.23 | 836.16 | 339.79 |
| S104 | 1 | 1 | 4 | 45 | 57.09 | 20.70 | 30.66 | 89.70 | 224 | 106 | 40 | 144.6 | 22929.85 | 4023.05 | 5281.67 | 296.13 | 1409.41 | 2002.01 | 828.93 | / |
| S105 | 1 | 4 | 2 | 25 | 53.37 | 24.38 | 26.58 | 32.91 | 201 | 93 | 26 | 134.6 | 23361.23 | 6318.67 | 11285.41 | 1542.44 | 4126.83 | 5189.39 | 901.35 | 538.35 |
| S106 | 1 | 3 | 2 | 25 | 53.92 | 24.34 | 26.98 | 37.28 | 203 | 92 | 36 | 134.6 | 18193.10 | 3248.48 | 5609.94 | / | 984.95 | 2125.82 | 524.87 | / |
| S107 | 2 | 3 | 4 | 45 | 56.55 | 21.79 | 28.71 | 63.36 | 224 | 105 | 39 | 155.1 | 22350.59 | 2327.86 | 5971.70 | 307.67 | 1611.36 | 1793.96 | 563.54 | 701.90 |
| S108 | 2 | 3 | 4 | 45 | 56.36 | 21.65 | 28.49 | 60.46 | 225 | 106 | 37 | 155.1 | 22629.15 | 1992.93 | 6792.97 | 531.35 | 1330.39 | 2006.22 | 439.86 | / |
| S109 | 2 | 3 | 4 | 45 | 49.54 | 24.40 | 21.35 | 5.80 | 220 | 100 | 33 | 118.2 | 26667.56 | 2401.12 | 6011.69 | 316.30 | 1810.38 | 1610.54 | 1166.43 | / |
| S110 | 1 | 3 | 4 | 45 | 49.39 | 24.29 | 21.19 | 5.58 | 211 | 96 | 35 | 118.2 | 26818.91 | 5852.71 | 9099.89 | 288.26 | 2317.39 | 4799.63 | 1572.81 | 680.45 |
| S111 | 1 | 3 | 4 | 45 | 49.69 | 24.12 | 22.09 | 7.41 | 205 | 93 | 33 | 129.7 | 17053.28 | 3415.72 | 6033.92 | 330.17 | 1852.23 | 5669.73 | 1790.88 | / |
| S112 | 2 | 3 | 2 | 25 | 50.36 | 24.03 | 22.57 | 8.09 | 223 | 106 | 26 | 129.7 | 21612.70 | 2348.79 | 3570.21 | / | 950.72 | 827.93 | 621.36 | 572.94 |
| S113 | 2 | 3 | 4 | 45 | 50.84 | 19.18 | 23.16 | 18.02 | 214 | 97 | 27 | 124.6 | 25941.96 | 4376.84 | 6011.47 | / | 1427.27 | 2218.39 | / | 623.73 |
| S114 | 2 | 3 | 4 | 45 | 51.80 | 19.48 | 24.08 | 21.18 | 216 | 107 | 33 | 124.6 | 19737.46 | 3963.11 | 4076.68 | 448.04 | 1740.66 | 3932.49 | 1304.39 | 545.62 |
| S115 | 4 | 2 | 4 | 45 | 47.50 | 21.74 | 18.37 | 9.01 | 156 | 62 | 6 | 135.6 | 15064.69 | 2936.21 | 5445.70 | 336.64 | 1046.58 | 1830.54 | 806.21 | 852.53 |
| S116 | 4 | 2 | 4 | 45 | 47.03 | 21.59 | 18.02 | 11.42 | 164 | 64 | 5 | 135.6 | 16674.75 | 3784.48 | 6168.9 | 375.39 | 1774.38 | 2658.40 | 781.19 | 647.68 |
| S117 | 4 | 2 | 4 | 45 | 51.44 | 25.61 | 24.61 | 19.80 | 176 | 73 | 7 | 134.1 | 15317.84 | 2719.56 | 3963.48 | / | 1521.71 | 2343.6 | 757.98 | 463.28 |
| S118 | 4 | 2 | 4 | 35 | 51.74 | 25.54 | 24.68 | 20.09 | 155 | 62 | 8 | 134.1 | 14762.27 | 3544.20 | 5255.65 | / | 1230.67 | 2300.41 | 625.21 | 490.53 |
| S119 | 4 | 2 | 4 | 45 | 51.83 | 25.69 | 24.97 | 22.23 | 163 | 68 | 9 | 123.9 | 14698.48 | 3404.60 | 5150.13 | / | 1201.95 | 2306.12 | 740.74 | 966.14 |
| S120 | 4 | 2 | 4 | 45 | 51.81 | 25.71 | 24.96 | 22.21 | 168 | 70 | 5 | 123.9 | 15146.64 | 3305.83 | 5312.65 | / | 1219.14 | 2572.75 | 902.31 | 909.50 |
| S121 | 4 | 2 | 4 | 45 | 47.19 | 21.96 | 18.95 | 9.91 | 167 | 72 | 3 | 118.4 | 15482.62 | 3525.17 | 5852.16 | / | 1343.76 | 2320.30 | 867.52 | 979.05 |
| S122 | 4 | 2 | 4 | 45 | 47.38 | 21.94 | 18.99 | 9.15 | 164 | 71 | 8 | 118.4 | 14210.33 | 3461.65 | 5574.89 | / | 1325.16 | 2354.47 | 636.22 | 968.62 |
| S123 | 4 | 2 | 4 | 45 | 49.21 | 20.12 | 20.42 | 8.72 | 182 | 82 | 8 | 124.4 | 16019.48 | 3297.79 | 5751.24 | / | 1355.78 | 2348.90 | 860.83 | 917.40 |
| S124 | 2 | 2 | 4 | 45 | 49.22 | 20.14 | 20.34 | 8.51 | 225 | 122 | 39 | 124.4 | 16467.83 | 3380.35 | 4850.53 | 582.47 | 1499.13 | 1814.46 | 395.53 | 203.03 |
| S125 | 2 | 2 | 4 | 45 | 52.78 | 23.53 | 27.19 | 36.29 | 220 | 108 | 38 | 142.5 | 22364.29 | 3461.48 | 7034.27 | 1966.96 | 1513.57 | 3084.00 | 509.58 | 807.93 |
| S126 | 3 | 2 | 4 | 35 | 53.04 | 23.53 | 27.41 | 38.54 | 238 | 130 | 57 | 142.5 | 12900.05 | 1927.90 | 2814.25 | 682.60 | 942.63 | 1272.64 | / | 257.90 |
| S127 | 2 | 2 | 2 | 25 | 49.39 | 23.81 | 21.78 | 6.78 | 203 | 90 | 33 | 133.3 | 15493.10 | 5003.25 | 9549.56 | 1112.89 | 1274.76 | 5090.98 | 787.12 | 1415.97 |
| S128 | 1 | 2 | 2 | 25 | 49.45 | 23.83 | 21.89 | 7.01 | 218 | 101 | 37 | 133.3 | 15628.18 | 3595.29 | 5809.76 | 817.91 | 2125.42 | 6054.35 | 2008.18 | 418.03 |
| S129 | 4 | 2 | 2 | 25 | 50.06 | 23.13 | 22.94 | 9.62 | 203 | 101 | 31 | 118.1 | 7481.41 | 1354.78 | 6189.49 | 1833.70 | 1019.22 | 7549.57 | 540.34 | 409.04 |
| S130 | 3 | 1 | 2 | 25 | 50.06 | 23.20 | 23.08 | 10.21 | 220 | 108 | 48 | 118.1 | 24991.50 | 9390.45 | 14035.45 | 912.92 | 4501.55 | 6972.47 | 801.02 | 1253.37 |
| S131 | 1 | 1 | 4 | 35 | 49.27 | 24.27 | 21.98 | 8.03 | 222 | 106 | 39 | 124.5 | 27349.85 | 5093.56 | 7456.61 | 577.07 | 1849.88 | 3717.62 | 344.71 | 561.33 |
| S132 | 2 | 1 | 4 | 35 | 49.22 | 24.33 | 22.03 | 8.36 | 211 | 96 | 30 | 124.5 | 27398.02 | 4711.06 | 6678.14 | 350.44 | 1988.76 | 3759.19 | 31.30 | 718.79 |
| S133 | 2 | 1 | 4 | 35 | 50.34 | 23.48 | 23.63 | 12.41 | 195 | 95 | 29 | 112.7 | 15052.42 | 4392.92 | 6026.75 | 363.29 | 2736.70 | 6966.71 | 198.20 | 1016.52 |
| S134 | 3 | 1 | 5 | 30 | 50.50 | 23.52 | 23.87 | 13.44 | 208 | 97 | 38 | 112.7 | 18779.22 | 2812.21 | 5205.05 | / | 1046.96 | 1827.72 | 357.47 | / |
| S135 | 2 | 1 | 5 | 30 | 50.79 | 24.81 | 24.84 | 19.81 | 231 | 115 | 42 | 136.1 | 29464.12 | 8106.27 | 9614.45 | 320.61 | 3214.88 | 3705.73 | 1408.93 | 1355.26 |
| S136 | 1 | 1 | 5 | 30 | 50.77 | 24.79 | 24.74 | 19.19 | 218 | 106 | 43 | 136.1 | 24681.12 | 8203.97 | 7864.46 | 329.23 | 2350.35 | 3084.16 | 1107.27 | 770.71 |
| S137 | 3 | 1 | 5 | 30 | 55.23 | 24.28 | 28.47 | 54.63 | 202 | 91 | 29 | 130.1 | 25842.40 | 4560.72 | 5956.70 | / | 2424.06 | 2942.42 | 947.03 | 369.19 |
| S138 | 1 | 1 | 4 | 45 | 55.23 | 24.31 | 28.47 | 54.66 | 214 | 95 | 33 | 130.1 | 27270.79 | 5865.83 | 5924.75 | / | 1880.21 | 2466.38 | 854.33 | 626.52 |
| S139 | 2 | 1 | 4 | 45 | 50.39 | 24.33 | 23.47 | 12.15 | 200 | 84 | 23 | 139.0 | 25126.75 | 5342.23 | 5635.01 | / | 1478.32 | 1699.96 | 893.69 | 388.02 |
| S140 | 3 | 1 | 4 | 45 | 50.31 | 24.20 | 23.31 | 11.37 | 225 | 108 | 38 | 139.0 | 27283.04 | 5738.78 | 7057.84 | 446.95 | 1672.50 | 2378.05 | 908.91 | 565.41 |
| S141 | 1 | 1 | 4 | 45 | 50.55 | 24.44 | 23.68 | 13.13 | 207 | 108 | 32 | 126.9 | 20846.6 | 2042.86 | 5825.58 | 1298.85 | 1268.32 | 2130.41 | 328.03 | 565.59 |
| S142 | 3 | 1 | 4 | 45 | 50.76 | 24.40 | 23.88 | 13.92 | 221 | 114 | 43 | 126.9 | 22602.67 | 3735.19 | 5070.19 | 420.26 | 1499.80 | 2151.19 | 960.79 | 272.33 |
| S143 | 1 | 1 | 4 | 45 | 49.51 | 23.97 | 22.18 | 7.93 | 214 | 98 | 39 | 134.8 | 23797.51 | 3736.10 | 5034.04 | 303.09 | 1484.17 | 1983.38 | 839.21 | 347.06 |
| S144 | 1 | 1 | 4 | 45 | 49.84 | 24.20 | 22.47 | 8.56 | 224 | 106 | 40 | 134.8 | 22935.59 | 4055.32 | 5155.76 | / | 1410.94 | 2006.11 | 814.11 | / |

Grade of quality (Q) ;storage life ( N ); drying method (M ); drying temperature (T ); color values ( L *, a *, b *, ΔE * ) measured by spectrophotometer; color values ( R, G, B ) and brightness ( Brightness, LD ) calculated based on RGB; HSYA is Hydroxysafflor yellow A; AHSYB is Anhydrosafflor yellow B; HKT is 6-Hydroxykaempferol-3,6,7-tri-O-glucoside; HKR is 6-Hydroxykaempferol-3-O-rutoside-6-O-glucoside; HKD is 6-Hydroxykaempferol-3,6-di-O-glucoside; HAG is 6-Hydroxyapigenin-6-O-glucoside-7-O-glucuronic acid; KR is Kaempferol-3-O-rutoside ; KD is Kaempferol-3,7-di-O-glucoside. “/” indicates no detection.
